# A Nuclear Factor of High Mobility Group Box Protein in *Toxoplasma gondii*

**DOI:** 10.1371/journal.pone.0111993

**Published:** 2014-11-04

**Authors:** Hui Wang, Tao Lei, Jing Liu, Muzi Li, Huizhu Nan, Qun Liu

**Affiliations:** Key Laboratory of Animal Epidemiology and Zoonosis, Ministry of Agriculture, National Animal Protozoa Laboratory, College of Veterinary Medicine, China Agricultural University, Beijing, China; Pasteur Institute Lille, France

## Abstract

High mobility group box 1 (HMGB1) is a nuclear factor that usually binds DNA and modulates gene expression in multicellular organisms. Three HMGB1 orthologs were predicted in the genome of *Toxoplasma gondii*, an obligate intracellular protozoan pathogen, termed TgHMGB1a, b and c. Phylogenetic and bioinformatic analyses indicated that these proteins all contain a single HMG box and which shared in three genotypes. We cloned TgHMGB1a, a 33.9 kDa protein that can stimulates macrophages to release TNF-α, and, we demonstrated that the TgHMGB1a binds distorted DNA structures such as cruciform DNA in electrophoretic mobility shift assays (EMSA). Immunofluorescence assay indicated TgHMGB1a concentrated in the nucleus of intracellular tachyzoites but translocated into the cytoplasm while the parasites release to extracellular. There were no significant phenotypic changes when the TgHMGB1a B box was deleted, while transgenic parasites that overexpressed TgHMGB1a showed slower intracellular growth and caused delayed death in mouse, further quantitative RT-PCR analyses showed that the expression levels of many important genes, including virulence factors, increased when TgHMGB1a was overexpressed, but no significant changes were observed in TgHMGB1a B box-deficient parasites. Our findings demonstrated that TgHMGB1a is indeed a nuclear protein that maintains HMG box architectural functions and is a potential proinflammatory factor during the *T.gondii* infection. Further studies that clarify the functions of TgHMGB1s will increase our knowledge of transcriptional regulation and parasite virulence, and might provide new insight into host–parasite interactions for *T. gondii* infection.

## Introduction

High mobility group box 1 (HMGB1) was first discovered in calf thymi as a nuclear protein that contained a unique DNA-binding domain and showed rapid migration in polyacrylamide gels, a property of the HMG superfamily. The two HMG-box containing HMGB proteins are only present in multicellular animals, and the HMGB gene apparently arose through the fusion of two different genes, each coding for one of the boxes [1]. Mammalian HMGB1 encodes 219 amino acids (aa) and contains two DNA-binding motifs (A-box and B-box) that are arranged in tandem, following a long negatively charged C-terminus that is rich in aspartic and glutamic acids, which differ in length (HMGB1–3) or are absent (HMGB4) [2]. By contrast, yeast HMGBs (Nhp6-a and -b) have only one HMG box domain and no acidic tail [3]. HMG proteins can bind to cruciform, double- and single-stranded DNA with high affinity through HMG-box and acidic C-terminus [4], [5]. Interactions between DNA and HMGs are mediated by basic amino acid residues of the protein. Structural studies using nuclear magnetic resonance spectroscopy established that the DNA binding domain of HMG has an L-shaped structure made of three α-helices that provide surfaces for potential interactions with both DNA and protein [2], [6].

In higher organisms, HMGBs are ubiquitously expressed in cell nuclei and act as DNA chaperones that influence multiple processes in chromatin, such as transcription, replication, recombination, DNA repair and genomic stability [7]. Variety of post-translational modifications (PTMs) such as acetylation, phosphorylation and methylation to HMGB can modulate not only HMGB1 protein function but also its subcellular location and eventual secretion [8]. HMGB1 is a prototypical damage-associated molecular pattern and the only one of these proteins that can be passively and actively secreted into the extracellular milieu, where it acts as cytokines, chemokines and growth factors that promotes cell migration and inflammation [9], [10], [11].

HMGB1 is highly conserved with >98% amino acid identity between humans and rodents [12], but appears to be more polymorphic among parasite species. HMGB proteins have been reported in many parasites including *Wuchereria bancrofti*, *Brugia malayi* [13], *Trypanosoma cruzi* [14], [15], *Schistosoma mansoni* [16], *Schistosoma japonicum* [17], *Plasmodium falciparum* [18], [19], [20], *Entamoeba histolytica* [21] and *Babesia bovis* [22]. Mammalian HMGB1 bears two DNA binding motifs followed by an acidic C-terminus, whereas most HMGB1 family members of parasites have only one or more HMG box and no acidic tail.

*T. gondii* is an obligate intracellular protozoan that can actively invade almost all nucleated cells and can cause opportunistic disease in various animals and humans [23]. The pathological basis for Toxoplasmosis is tissue destruction and inflammation, which are a direct result of the parasite's cell lytic growth cycle of attachment, invasion, growth, and egress. There are three *T. gondii* genotypes, types I, II, and III, which have different growth characteristics [24] and cause variable levels of virulence in mice [25], [26]. Type I strains are uniformly lethal at all doses (high virulence) in all strains of laboratory mice, whereas type II (intermediate virulence) and type III (low virulence) show much lower levels of pathogenicity. Virulence in type I strains is a result of rhoptry effector proteins (ROP18, ROP5 and ROP16) effectively eliminating critical host immune responses which leads to uncontrolled proliferation of the tachyzoite, and host survival is compromised due to excessive parasite burden. Type II strains induce stronger proinflammatory responses, including very high levels of IL-12 in comparison with either type I or III and the susceptible animals always die of severe inflammation. Like type I, Type III strains limit the initial production of pro-inflammatory cytokines, whereas be unable to avoid intracellular killing mediated by IRGs and late production of IL-12 by DCs triggers a Th1-type response that is sufficient to control parasite burden and induce cyst formation, leading to a chronic infection (reviewed by [27]). The transitions between the different stages of the *T. gondii* life cycle allow the parasite to be virulent and survive. These developmental transitions are accompanied by major changes in gene expression [28], and the control mechanisms for parasite proliferation (replication and differentiation) may be regulated by the cell cycle [29], [30] and the micro-environments around the parasites [31]. Regulation of *T. gondii* gene expression is, in part, promoted by epigenetic events, such as histone modifications and interactions between histones and other nuclear factors [32].

In ToxoDB earlier version (version 9.0, http://toxodb.org/toxo/), several genes were predicted to encode HMG proteins in *T. gondii* genome, though there are many information even including the coding sequence were updated in the latest *T.gondii* genomic database (ToxoDB version 11), the characteristics of these protein, such as subcellular locations and biological functions are poorly understood to date. Therefore, we aimed in this work to identify and characterize HMGB homologues in *T. gondii* and also to assess whether HMGB proteins relate to gene regulation as an architectural factor and contribute to parasite growth or virulence to mice.

## Material and Methods

### Ethics statement

All experiments with animals in this study were performed in strict accordance with the recommendations in the Guide for the Care and Use of Laboratory Animals of the Ministry of Science and Technology of China. Formal animal ethics of all experimental procedures were approved by the Institutional Animal Care and Use Committee of China Agricultural University (The certificate of Beijing Laboratory Animal employee, Approval No: 18049). All efforts were made to minimize animal suffering.

*Toxoplasma* culture using HFF (human foreskin fibroblasts) monolayers are generally appeared in many papers about *Toxoplasma* (for example: John C. Boothroyd, Pernas L, Adomako-Ankomah Y, Shastri AJ, Ewald SE, Treeck M, et al. (2014) *Toxoplasma* effector MAF1 mediates recruitment of host mitochondria and impacts the host response. PLoS Biol 12: e1001845.). In this study, we used the HFF cell to maintain *T.gondii* only, which is a normal system for culture the parasites in vitro. HFF cell line was bought from the ATCC company (USA) and serial passaged in our lab. From the company websites, products reference: Hovatta O, et al. A culture system using human foreskin fibroblasts as feeder cells allows production of human embryonic stem cells. Hum. Reprod. 18: 1404–1408, 2003. PubMed: 12832363.

### Parasites and cell culture

HFF (human foreskin fibroblasts) were cultured in complete DMEM, as described previously [33]. *T. gondii* tachyzoites were maintained in vitro by serial passage on confluent HFF monolayers in DMEM containing 25 mM glucose and 4 mM glutamine supplemented with 10% fetal bovine serum (FBS, Gibco, USA), and incubated at 37°C with 5% CO_2_ in a humidified incubator. The medium was changed 6 hours after inoculation.

### Sequence alignment and molecular phylogenetic analyses of HMGB1s

The sequences used in this study were obtained from the EuPathDB database and National Center for Biotechnology Information (NCBI) GenBank (**Table S1**). Phylogenetic analyses were performed based on either the Neighbor-Joining method [34] using the substitution model with partial deletion of gap containing sites (1000 bootstrap replicates were performed) or the Maximum Likelihood (ML) method with an approximate likelihood-ratio test and JTT selected for amino acid substitution model analysis with a CNI search to start the initial tree [35]. All alignments were performed using DNAMAN version 5.2.2 and ClustalX2, and phylogenetic trees were visualized with Mega 5.22 [36]. Bootstraps values obtained from the phylogenetic analyses are indicated on trees presented in **Figure S1**. HMGB1 proteins from different organisms were considered to be significant, allowing clusters to form that are represented by colored clades.

### Bioinformatic analyses of the TgHMGB1s sequence

A BlastP search using the complete sequence of *Mus musculus* High mobility group box 1 deposited in GenBank (accession no. AAH83067.1) was performed using ToxoDB (http://toxodb.org/toxo/, ver.9.0) within the *Toxoplasma* GT1 database. This search identified three putative transcripts with significant similarity to HMGB1 protein, termed TgHMGB1a, TgHMGB1b and TgHMGB1c. The molecular weight, isoelectric point, amino acid composition and putative protein functions were also predicted using BlastP. The presence of a HMG-box was confirmed using the Conserved Domain Search Service (CD Search) from NCBI. Also, characteristics of the primary sequence of TgHMGB1a were analyzed using the following online servers: (1) signal peptide cleavage sites in the TgHMGB1a sequence were predicted using Signal IP4.1 (http://www.cbs.dtu.dk/services/SignalP/); (2) transmembrane regions and the orientation of TgHMGB1a were analyzed using TMHMM Server v. 2.0 (http://www.cbs. dtu.dk/services/TMHMM-2.0/); (3) subcellular localization (http://wolfpsort.org/) and nuclear localization signal prediction (https://rostlab.org/owiki/index.php/PredictNLS); (4) to identify conserved domains, online tools (http://myhits.isb-sib.ch/cgi-bin/motif_scan; http://www.expasy. org/tools/scanprosite/) were used to search for motifs and conduct additional bioinformatics analyses; (5) sequence alignment analysis and three-dimensional (3D) structure modeling performed using ESPript 2.2 (http://espript.ibcp.fr/ESPript/cgi-bin/ESPript.cgi) and homology remodeling tools (http://zhanglab.ccmb.med.umich.edu/I-TASSER/; http://swissmodel.expasy.org/workspace/index; and http://www.cbs.dtu.dk/services/CPHmodels/). Software programs such as DNAstar, DNAMAN and Primer Premier 5 were also used in the analyses.

### Cloning and identification of TgHMGB1a

Based on the gene sequence of TgHMGB1s, overlapping primers (**Table S2**) were designed to amplify the complete coding sequence of TgHMGB1a from the RH strain cDNA prepared from total RNA using oligo (dT) primers. Overlapping PCR reactions and touchdown PCR programs were applied to gain high fusion rates throughout the process, and the resultant products were cloned into a pEASY-T-Blunt vector (Beijing TransGen Biotech Co., Ltd.). Finally, the DNA insert was sequenced and analyzed by Blast to confirm the authenticity of the cloned sequence. Then a truncated TgHMGB1a fragment (including the B box but not the transmembrane domains) was amplified using specific primers (TgHMGB1a 4E-F and -R listed in **Table S2**). The products were digested (*Bam*HI and *Xho*I, NEB, Beijing), cloned into the pET-28a vector (Novagen, Germany), and transformed into *Escherichia coli* (*E.coli*) for expression. Only the soluble rTgTHMGB1a 4E were purified by affinity chromatography using Ni-IDA agarose (QIAGEN, Germany) following the manufacturer's instructions. Protein purity was assessed using SDS-PAGE and identified by western blot using the *T.gondii* positive pig serum.

Normal immune procedure of the Freund's Adjuvant (Sigma) were used to raise antibodies against rTgTHMGB1a 4E in Balb/c mice. Anti-rTgH1a4E sera were collected 2 weeks after the last immunization. The specificities and titers of polyclonal antibodies were examined using immunoblotting and enzyme-linked immunosorbent assays (ELISA), respectively, using conventional protocols. All sera samples were sterilized by filtration through 0.22 µm filters (Millipore, USA) and stored at −80°C.

### Immunofluorescence assays and western blotting

Immunofluorescence assays (IFA) for TgHMGB1a subcellular localization were performed as described previously [37]. Briefly, freshly released RH stain *T. gondii* were passed through 5 µm filters, spun down by centrifugation at 300×*g* for 10 minutes, and resuspended using PBS. Parasites were seeded at appropriate amounts onto confluent monolayers of HFF cells grown on previously prepared glass coverslips in 12-well plates. Infected cells were incubated at 37°C in 5% CO_2_ for 16–20 hours and the fixed for 15 min in 3.7% formaldehyde. Cells were next permeabilized with 0.3% Triton X-100 for 10–15 min, and blocked with 3% bovine serum albumin (BSA) in phosphate-buffered saline (PBS) for 30 minutes prior to incubation with the primary antibody (diluted 1∶100 in 1% BSA-PBS) for 1 hour. Coverslips were washed three times in PBS and then incubated with FITC-conjugated goat anti-mouse IgG (H+L) that was diluted 1∶100 in PBS with 1% BSA (Proteintech, USA) for 1 hour at 37°C. Nuclei was stained with Hoechst 33258 (Sigma) and coverslips were subsequently mounted onto microscope slides. Fluorescence images were obtained with a Leica microsystem (Leica TCS SP5 II, Germany) and epifluorescence optics using an oil immersion lens with 63× magnification. Collected images were processed using LAS AF lite 2.2.0 software (Leica). Rabbit anti-MIC3 antibodies and preimmune mouse sera were used as controls. To analyze TgHMGB1a in extracellular parasites, filtered parasites were allowed to adhere onto the coverslips precoated with poly-lysine (poly-L-Lys) for 20 minutes at room temperature and were then processed similarly to intracellular parasites.

Purified parasites were lysed using RIPA buffer (Beyotime, Beijing) with a cocktail of protease inhibitors, and 5 µg of lysate was loaded per lane for fractionation by 12% (w/v) SDS-PAGE. After electrophoresis, separated strips were transferred onto polyvinylidene fluoride (PVDF) membranes (Millipore, USA) together with a visible prestained protein marker. Membranes were blocked with 5% (w/v) skim milk in PBS for 1 h at 37°C, and then incubated with the mouse anti-rTgHMGB1a 4E antibody (diluted 1∶600 in PBS with 3% BSA). After three rounds of extensive washing in PBST (1% Tween-20), membranes were incubated for 1 h with goat anti-mouse IgG (H+L) horseradish peroxidase (HRP)-labeled secondary antibody (Sigma, USA) diluted 1∶10,000 in PBS with 3% BSA. Finally, reactive bands were visualized using enhanced chemiluminescence reagents (Co Win Biotech Co., Ltd., Beijing).

### Examination of TNF-α release

Peptides derived from TgHMGB1a B box, mouse HMGB1, mouse HMGB1 A box, mouse HMGB1 B box or a chimeric protein of mouse HMGB1 A box fused to TgHMGB1a B box were expressed in *E. coli*. Following sonication, recombinant proteins were purified as previously mentioned. Then, gel retardation experiments were carried out to test whether or not HMG peptides bind the DNA from *E. coli* genome. As the manual of molecular cloning, the natural and denatured (0.1% SDS) purified complexes of protein and DNA were resolved by electrophoresis on 1.5% agarose gels in 1× TBE buffer at 120 V for 15 minutes. DNA was stained with 0.5 µg mL^−1^ gold views (TransGen Biotech Co., Ltd., Beijing) and photographed using a UV transilluminator system. To evaluate the ability of TgHMGB1s stimulates to TNF-α release, murine peritoneal macrophages ana1 cell line were culture and stimulated with the recombinant peptides at indicated concentrations, and the culture medium was assayed for TNF-α at 24 h after stimulation. All of the recombinants were treated with Polymyxin B agarose (Sigma-Aldrich) twice and endotoxin contents were examined using the Limulus amebocyte lysate (LAL) kit (Lonza, USA), and only if the endotoxin content of proteins less than 0.06 EU/mg, it can be used to do the stimuli tests. The levels of TNF in the culture medium were determined and calculated using a commercial ELISA kit (Dakewe Biotech Co., Ltd., Beijing) following the instructions of manufacturer.

### Electrophoretic mobility shift assay (EMSA) with synthetic cruciform DNA

The partially complementary oligonucleotides 4H-a to 4H-d for EMSA corresponded to previous reports [14], [38] were synthesized to create cruciform DNA (4H) as well as “hairpin-like” structures (2H), by annealing the required oligonucleotides of equal amount in each case according to the instruction of EMSA Probe Biotin Labeling Kit (Beyotime, Beijing). Double-stranded (ds) linear DNA was obtained by annealing oligonucleotides 4H-a and 4H-c with corresponding fully complementary ones (Compl 4H-a and Compl 4H-c). Biotin-labeled DNA structures were obtained by annealing the biotin-labeled oligonucleotides labeled through the Terminal Deoxynucleotidyl Transferase (EMSA Probe Biotin Labeling Kit, Beyotime, Beijing). EMSA reactions were set up as follows (10 µL): 2.0 µL 5×DNA-binding buffer, increasing amounts of recombinant TgHMGB1a 4E protein (0.01–1.5 mg/mL), 5 nmol of the labeled cruciform DNA and add ddH_2_O to final volume. The reaction volumes were incubated at 25°C for 30 min. For competition assays, 100-fold or 500-fold molar excess of cold complete cruciform DNA (4H), incomplete cruciform DNA (2H) or linear dsDNA were added to the reaction and incubated for an additional 20 min at 25°C. Protein–DNA complexes were separated in 6% non-denaturing polyacrylamide gels in 0.5× Tris-borate-EDTA buffer (pH 8.0), run at 50 V at 4°C for 1.5 h. After electrophoresis, the bound and free probes in the gel were transferred to a positive charged nylon membrane (Millipore, USA) at 60 V at 4°C for 2 h. The probes on the membrane were detected using Chemiluminescent EMSA Kit (Beyotime, Beijing). Purified TgGRA1 proteins were used as control under the same conditions.

### Generation of TgHMGB1a B box-deficient parasites

A stable B box-deficient clone was established to further establish the function of TgHMGB1a. We constructed a gene targeting plasmid based on the single-step, O-type insertion strategy, as described in gene targeting protocols [39]. Briefly, 2200 bp of homologous sequence from a region upstream of TgHMGB1a B box and eGFP were fused using overlapping PCR (all primers used are shown in **Table S3**). The products flanked with *Avr*II and *Pac*I sites were introduced to the equivalent sites of pTCR (modified from pTCY), which we named the pTCR eGFP KO TgH1a B box plasmid. After digestion with *Bsi*WI (NEB, Beijing), linearized vectors were purified using ethanol and then resuspended using cytomix to transfect into the recipient RHΔKU80 strain. Parasites cultures and genetic manipulations were carried out according to the descriptions above, and chloramphenicol (CAT) and RFP were used as selection markers. A positive clone (i.e., a strain in which the B box was replaced by eGFP) should exhibit triple-positivity for chloramphenicol resistance, RFP and eGFP, and the targeting event in triple-positive clones was confirmed using PCR and western blotting. A stable mutant was cloned after selection by FCM sorting.

### Overexpression of TgHMGB1a

To further characterize the role of TgHMGB1a, we generated a transgenic RH parasite strain that overexpressed TgHMGB1a, and TgHMGB1a lacking the B box transgenic strain was also constructed as control. A plasmid for transfection was constructed as follows: the complete sequence of TgHMGB1a was amplified from a pEASY-T-TgHMGB1a plasmid using primers (**Table S3**) that appended the flanking *EcoR*V and *Nsi*I restriction endonuclease sites. Amplification products were introduced into the equivalent sites of a pDMG plasmid, and the resulting vector was used as an overexpression vector for transfection that we designated as pDMG-TgHMGB1a. The fused TgHMGB1a-GFP gene was under control of the *T. gondii* GRA1 promoter. The TgHMGB1a overexpression strain was generated using electroporation transfection [40] and selected based on pyrimethamine resistance as described previously [41]. Finally, flow cytometry (FCM) was used to acquire a monoclone. B box lacked TgHMGB1a overexpress strain (TgHMGB1a^−B box^) was generated as the same methods.

### Plaque assay and intracellular replication test

Plaque assays to compare the transgenic parasites with their parental strains were performed on HFFs cells in 6-well tissue culture plates (Corning costar, Beijing). Briefly, 500 parasites per well were seeded into confluent monolayers and infected cells were maintained in fresh DMEM containing 10% FBS and incubated undisturbed at 37°C in 5% CO_2_ for 7 days. To stain the monolayers, media was aspirated and disassociated parasites were washed off using PBS. Cell monolayers were then fixed for 10 minutes in PBS with 4% formaldehyde and stained with crystal violet solution (12.5 g crystal violet dissolved in 125 mL ethanol and mixed with 500 mL 1% ammonium oxalate in water) at room temperature for 10 minutes, washed with deionized water, air dried and visualized by microscopy using image acquisition and plaque area measurement as previously described [42].

To analyze the intracellular growth rate of the transgenic parasites compared with their parental strains, about 1×10^5^ parasites were inoculated on confluent HFFs in 24-well plates. The infected cells continuously incubated for 24 hours. Thereafter, cells were fixed in PBS with 4% formaldehyde, and RH and RHΔKU80 parasites were stained for IFA using rabbit anti-SAG1 polyclonal antibody as described above. TgHMGB1a overexpression and B box-deficient parasites were directly examined for fluorescence (GFP and eGFP, respectively). The numbers of parasites per vacuole for a minimum of 150 randomly chosen vacuoles were counted for each strain using a fluorescence microscope (IX71, Olympus, Japan) at 400× magnification.

### RNA isolation and real-time PCR

Total RNA was isolated from HFF cells that were uninfected or infected by either transgenic or parental parasites using the RNA isolation kit version 2.11 (Takara Biotechnology, Dalian, Co., Ltd) and treated with DNase I (Takara) to remove any residual genomic DNA. We synthesized cDNA using Oligo (dT)18 and random 6-mers according to the manufacturer's protocol for cDNA Synthesis Kit (Takara). Specific primers were designed using Primer Premier 5.0, including primers for TgHMGB1s (TgHMGB1b and TgHMGB1c), rhoptrys (ROP18, ROP16 and Toxofilin), micronemes (MIC3, PLP1), dense granules (GRA7), profilin and *T. gondii* actin (**Table S4**). The specificity of these primers was evaluated using conventional real-time (RT)-PCR. Quantitative RT-PCR (ΔΔCt method) was performed using the ABI Prism 7500 System (Applied Biosystems Inc. USA) with Tli RNaseH Plus kit (Takara). Resulting concentrations of RNA were normalized by TgActin and the relative expression levels of target genes were analyzed using ABI Prism 7500 software v2.0.5.

### Chromatin immunoprecipitation (ChIP) -qPCR analysis

ChIP assays was applied to investigate whether TgHMGB1a directly participate transcriptional regulation of the indicated genes in the wild type and the TgHMGB1a overexpress strain. ChIP assays were carried out according to the manufacturer's protocol of the ChIP Assay Kit (Beyotime, Beijing) with slight modifications. Briefly, the chromatin solutions from equal number of purified RH and TgHMGB1a overexpress strain were sonicated and immunoprecipitated with anti-TgHMGB1a 4E sera in rotating overnight at 4°C. Parallel controls for each experiment included samples with the normal mice IgG (negative control) and the input (positive control), and TgHMGB1a B box^−/eGFP^ parasites were also used as control. After elution and purification, the recovered DNA samples were used as template for regular PCR or real-time PCR performed using the ABI Prism 7500 System with Tli RNaseH Plus kit (Takara). The promoter-specific primers of indicated genes were listed at **Table S5,** and the coding region primers (**Table S4**) were used as controls. Pull-down level of the target promoter sequences were normalized to the corresponding abundance in the input chromatin. After amplification, regular PCR products were resolved on 1.5% agarose gel and visualized by gold view staining, while the quantitative-PCR were analyzed using the ABI Prism 7500 Software v2.0.5.

### Mouse infectivity studies

All the 8- to 10-wk-old female Balb/c mice were purchased from the Center for Experimental Animals (Beijing). Rodent laboratory chow and tap water were provided ad libitum and maintained under specific pathogen-free conditions for 7 days before manipulation. To assess the virulence of TgHMGB1a overexpress strain compared with the parental line, TgHMGB1a overexpress parasites, RH and RH-GFP tarchyzoites were injected intraperitoneally (i.p.) into mice at doses of 10^2^, 10^3^ and 10^4^ (5 mice per dose), and mice were monitored until there were no remaining survivors. Similarly, to examine the virulence of the TgHMGB1a B box deficient, TgHMGB1a B box^−/eGFP^ parasites were injected i.p. into mice at doses of 10^2^, 10^3^ and 10^4^ (5 mice per dose). In comparison, RHΔKU80 was injected into mice at the same doses (5 mice per dose). Mice were also monitored until there were no remaining survivors or for 30 d. All the infected mice were monitored three times a day (every 8 hours) for clinical signs and mortality until there were no remaining survivors. The mice were humanely euthanized when they were unable to reach food or water for more than 24 h and lost 20% normal body weight. The mice were humanely euthanized by cervical dislocation after anesthetization. The mice were anesthetized by subcutaneous injection of Atropine (0.02 mg/kg) before euthanasia. All procedures were in strict accordance with the PR China legislation on use. All efforts were made to minimize animal suffering. And a completed ARRIVE checklist for the animal experiments can be found in **Data S1**.

### Analysis of data and statistics

All data sets are presented as the mean ± SD and all statistical analyses were performed using SPSS 18.0 (USA). Unpaired two-tailed Student's *t*-tests with unequal variance were performed.

## Results

### Only one HMG box in *T. gondii* “high mobility group box” proteins

**Figure 1:**
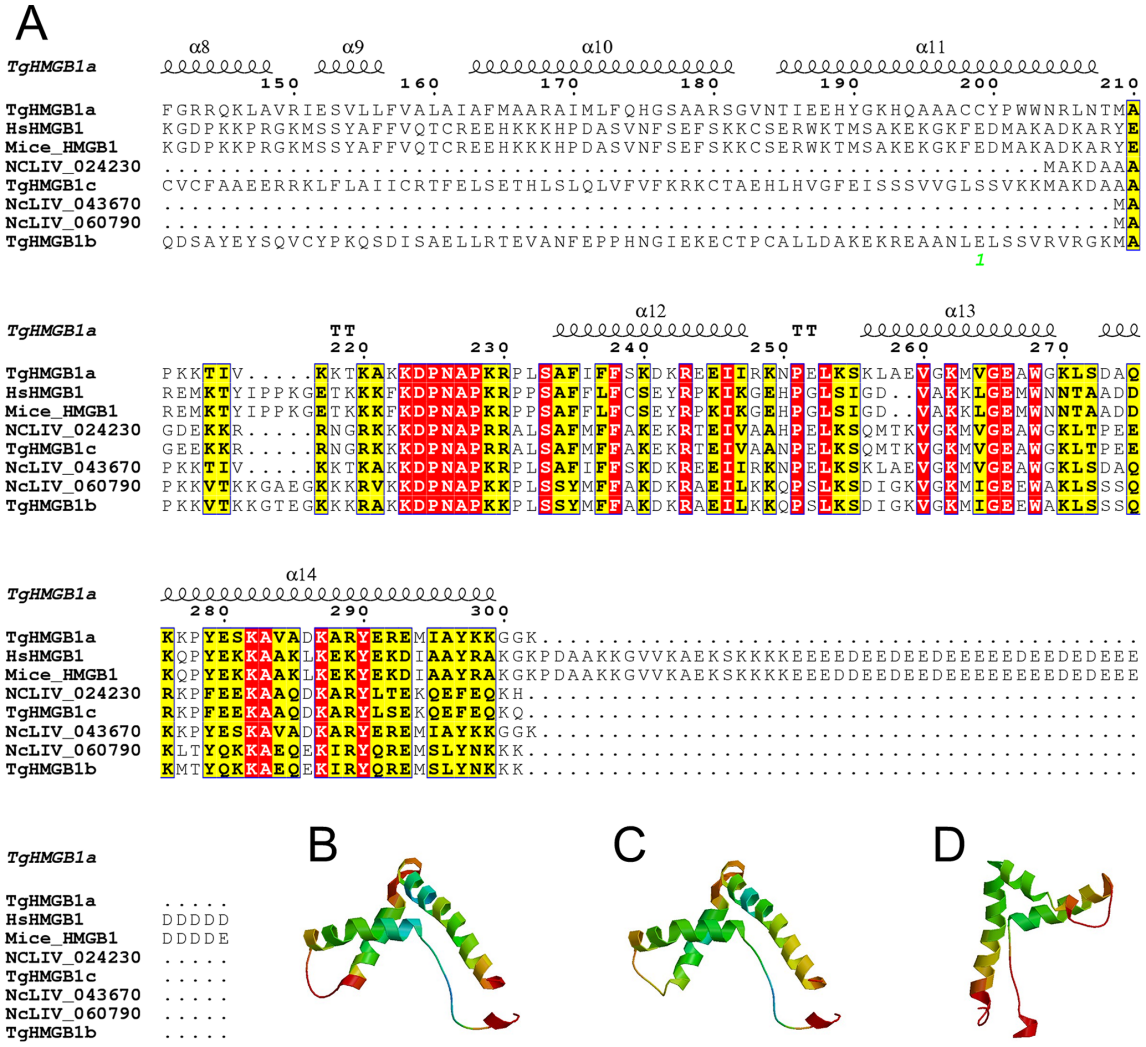
Sequence-structure alignment of HMGB1s and spatial structures prediction of HMG box in TgHMGB1a, b and c. A. *T. gondii* and *N. caninum* HMG box domains were identified using Motif Scan and are marked by a box and compared with sequences of human and mice. The positions of identical and conserved residues contained in the HMG box are indicated by red- and yellow-filled rectangular frames, respectively. Dots indicate gaps or missing residues. The secondary structure of TgHMGB1a was also predicted and α-helices 12, 13 and 14 represent the three α-helices of the TgHMGB1a HMG box. B, C, and D. A three-dimensional model of the HMG box in TgHMGB1a (B), TgHMGB1b (C) and TgHMGB1c (D) were built based on homology modeling. As shown here, the three α-helices fold into a characteristic L-shaped domain expected for a HMG box.

HMG box-containing proteins were identified in the three genotypes of *T. gondii* using the Blast sequence homology algorithm (**Table S6**). Here, TgHMGB1a, b, c were named to represent three HMGB1 proteins in the GT1 strain annotated in the ToxoDB database (corresponding to TGGT1_053220, TGGT1_030840 and TGGT1_043980 in ToxoDB ver.9.0, but renamed to TGGT1_210412, TGGT1_219832, TGGT1_263720 in ToxoDB ver.11, **Figure S1 and S2**), which encode 33.9, 74.1 and 20.1 kDa proteins, respectively. Phylogenetic analysis showed that TgHMGB1a, b and c could be classified into three significant clusters through both the neighbor-joining (NJ) and maximum likelihood (ML) algorithms (**Figure S1**), which most because for different conservation of HMG domain in their C-terminal. However, TGGT1_217500 may be impossible to assign specific homologues to this gene because of low bootstraps values. There were genes that were clearly orthologous between *T. gondii* and *N. caninum*. TgHMGB1a corresponds to NcLIV_043670, while TgHMGB1b and TgHMGB1c correspond to NcLIV_060790 and NcLIV_024230, respectively. Five HMG proteins were also predicted in *Eimeria. spp.* (**Figure S1**) in ToxoDB ver.11. The two HMGB genes of *P. falciparum* (PfHMGB1/2) mapped to the TgHMGB1b cluster, while *T. cruzi*, *T. brucei*, *S. mansoni*, and *L. major* HMGB1s all mapped to branches that were far away from the TgHMGBs. *S. cerevisiae* HMGB1s formed an independent branch that was near to the TgHMGB1s. The *A. thaliana*, *M. musculus* and *H. sapiens* HMG genes, HMGB1, HMGB2, HMGB3 and HMGB4, were also analyzed in this study. The HMGBs of vertebrate animals and plants were clearly branched in two significant clades, supporting the conserved evolution of these proteins. Unlike mammalian HMGB1, TgHMGB1 proteins have only one HMG box domain (∼71 aa) that is similar to the B box of *H. sapiens*, as box A and an acidic C-terminus are both absent (**Figure 1A** **and** **S2**). The isoelectric points (pI) of these three proteins were all close to 9.6, which is characteristic of many DNA binding proteins. The three-dimensional (3D) structures of the HMG box domains of TgHMGB1a, b and c were homology modeled with the SWISS-MODEL server using *M. musculus* 2gzkA as a template structure. The three α-helices in the C-terminal domain of TgHMGB1a, b and c fold into an L shape that formed the HMG box (**Figure 1B, C, and D**, respectively).

### TgHMGB1a is a typical one HMG box protein and concentrated in nuclear in intracellular tachyzoites but translocated into cytoplasm while it's in extracellular

**Figure 2:**
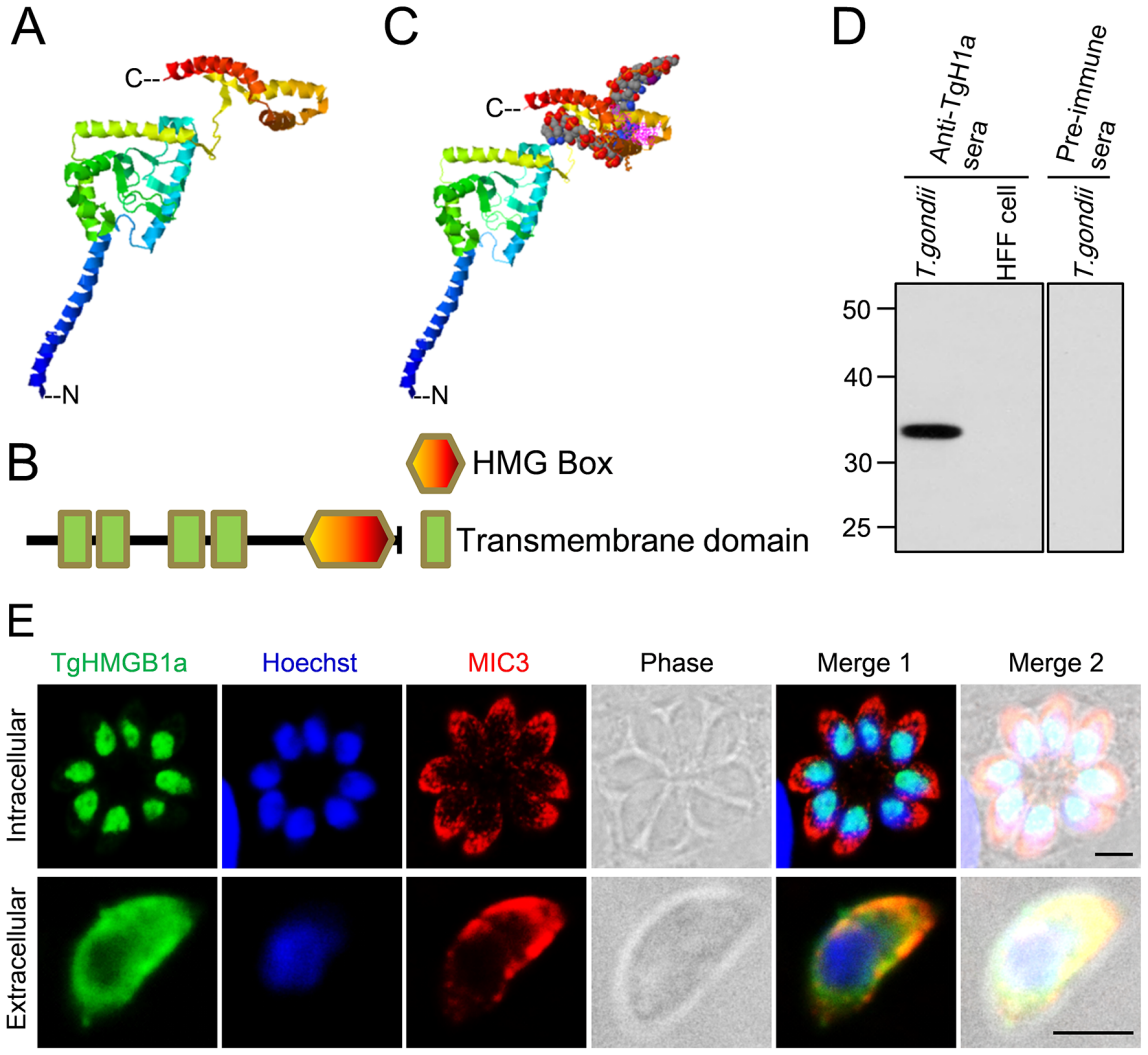
Characterization of TgHMGB1a by structure modeling and cellular localization. A. Ribbon cartoon representations of the predicted 3D structure of TgHMGB1a generated by homology modeling. Three α-helices independently fold into the HMG box at the C-terminal, while another four helices are clustered as the transmembrane domains. C indicated the C-terminal and N showed the N-terminal. B. Schematic representation of the primary structure of TgHMGB1a highlighting its different domains. TgHMGB1a has only one HMG box at the C-terminus and four transmembrane domains at the N- terminal; there is no acidic C-tail. C. A model of TgHMGB1a bound to DNA. TgHMGB1a can bind DNA through an interaction between the HMG box and the minor groove of DNA. D. Identification of native TgHMGB1a by western blotting. *T. gondii* represents the total antigens from cell cultured *T. gondii* tachyzoites; uninfected HFF cell lysates were used as a control. A specific band was elicited when anti-rTgHMGB1a 4E polyclonal antibodies were used as a primary antibody, but not when preimmune serum was used. E. TgHMGB1a is concentrated in the nucleus of parasite. Immunofluorescent localization of TgHMGB1a showed that TgHMGB1a (green) not only localized in the nucleus (blue) during cytokinesis (C) (top panel), but also during mitosis (M) (middle panels). Additionally, TgHMGB1a continued to accumulate in the nucleus of tachyzoites freshly released into extracellular media (bottom panel). MIC3 (red), nuclei stained with Hochest (blue). Scale bars, 3 µm. All immunofluorescent labeling was performed on HFF cells infected with tachyzoites of the RH strain.

The ternary structure of full length TgHMGB1a (**Figure 2**A) was modeled using the structure template of 1ckt A chain. TgHMGB1a encodes 302 aa and contains only one 71 aa HMG box (228–298 aa) that is independently folded at the C-terminus, and 4 upstream transmembrane helices (31–50, 60–82, 119–141 and 151–173 aa) (**Figure 2B** **and** **S3B**). As expected, the HMG box domains showed the typical three α-helices in an L-shaped fold (**Figure 1B** and **2A**). And eight residues (Ser233, Phe235, Leu257, Ala258, Gly261, Lys262, Gly265 and Trp268) were predicted to play a pivotal role in DNA binding ability. A model of a TgHMGB1a-DNA complex is shown in **Figure 2C**. All sequence analyses and structure predictions indicated that the TgHMGB1a is a DNA binding protein. Furthermore, classic signal peptide cleavage sites and nuclear localization signals were not found in TgHMGB1a (**Figure S3A**), but many residues involved in post-translational modifications (PTMs) were predicted (**Figure S3C**). The genomic DNA sequence annotation of TgHMGB1a in ToxoDB was 3391 bp, and the gene was interrupted by 3 introns. Using the fuse PCR program, 909 bp PCR products of TgHMGB1a coding sequence were obtained, and then confirmed by sequencing and alignment. Truncated TgHMGB1a (193–302 aa, termed rTgHMGB1a 4E, ∼14 kDa) was prokaryotic expressed as a His-tagged fusion protein and was strongly recognized by sera from swine infected with *T. gondii* (data not shown). Anti-rTgHMGB1a 4E sera was prepared from immunized mice that showed a high titer (10^6^) against rTgHMGB1a 4E. Immunoblotting showed that the preimmune sera did not react against *T. gondii* tachyzoite lysates, whereas mouse anti-rTgHMGB1a 4E antibodies elicited a 34 kDa protein band in *T. gondii* tachyzoite lysates, but not in HFF cell lysates (**Figure 2D**). Interestingly, the amount of TgHMGB1a was dramatically decreased in extracellular parasites, even almost undetectable while extending the time of extracellular (**Figure S4**).

Intracellular and extracellular RH strain tachyzoites were fixed and subjected to IFA using anti-rTgHMGB1a 4E antibodies to determine the subcellular location of TgHMGB1a (**Figure 2E**). In the up panel, TgHMGB1a (green) co-localized with Hoechst (blue), indicating that most of this protein concentrated into the nucleus in the intracellular RH strain tachyzoites (**Figure 2E** top panel), and even in the endodyogeny process, TgHMGB1a always locate in the nucleus (**Figure S5** top panels), but interestingly, while the parasites released to extracellular, the TgHMGB1a proteins do not remain in the nucleus, and have translocated into cytoplasm (**Figure 2E** bottom panel and **Figure S6** top panel), and dispersed around the cytoplasma membrane. For comparison, rabbit anti-TgMIC3 serum was used to stain microneme (denotes the cytoplasm) of parasites, and control experiments carried out with preimmune sera or secondary antibody alone showed no signal (data not shown). Additionally, the similar results were obtained when use the anti-SAG1 sera as the control (intracellular parasites showed in **Figure S5** bottom panel, while **Figure S6** bottom panels indicated the extracellular tachyzoites).

### TgHMGB1a is a DNA binding protein and induces TNF-α secretion in macrophages

**Figure 3:**
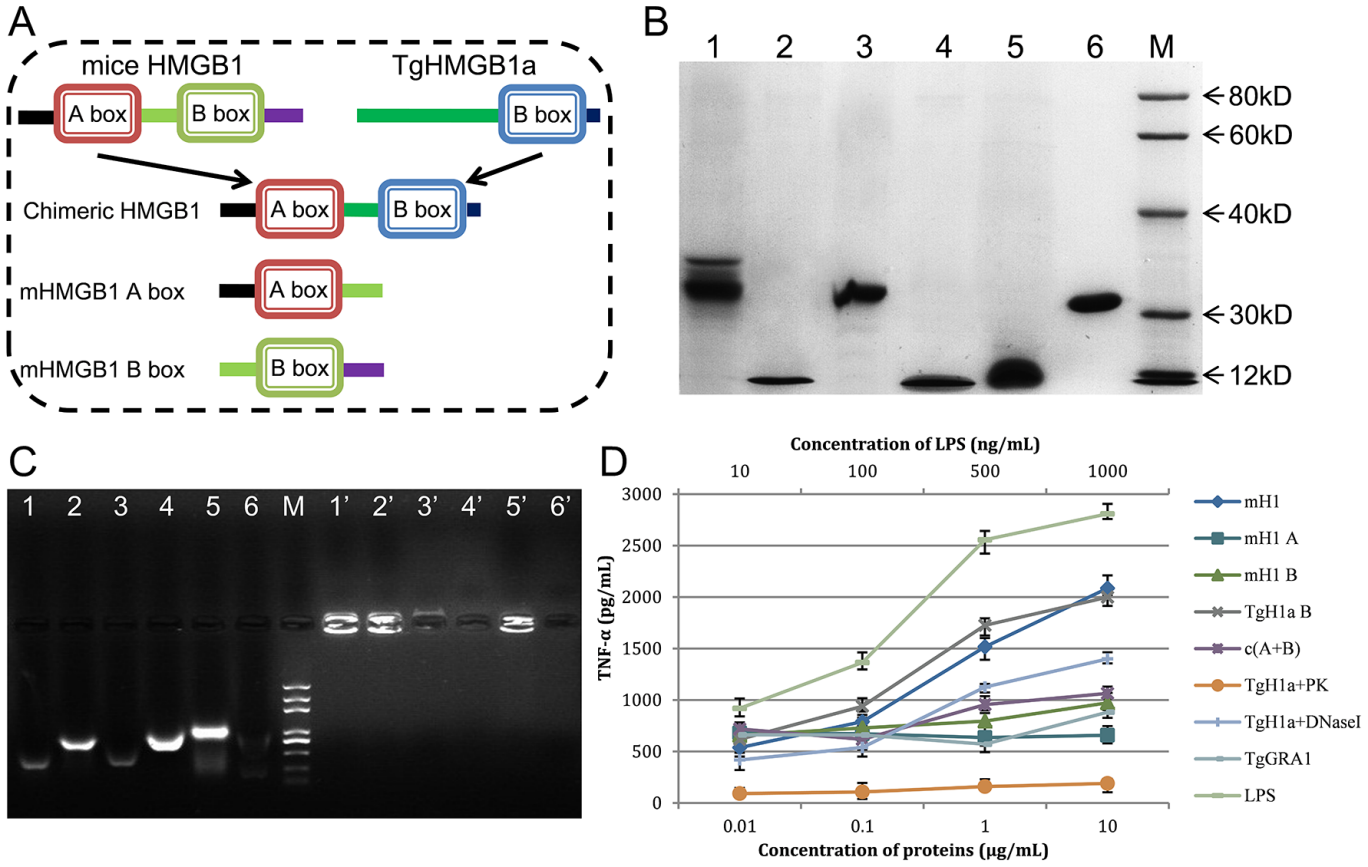
The TgHMGB1a box bound DNA fragments from *E. coli* and stimulated macrophages to release TNF-α. A. To examine TgHMGB1a DNA binding ability and to compare that with mouse HMGB1, full length mouse HMGB1 (mH1), TgHMGB1a 4E (TgH1a B), independent mH1 A and B box domains and a chimeric mH1 A box-TgH1a B box (c (A+B)) were expressed in the prokaryote *E. coli*. B. Purified recombinant peptides were detected by SDS-PAGE. Lane 1, mH1; lane 2, TgH1a B; lane 3, chimeric c(A+B); lane 4, mH1 A; lane 5, mH1 A; and lane 6, TgGRA1 (control). M indicates the protein marker. C. Gel retardation experiments were carried out to identify bound DNA fragments. Lanes 1–6: denatured DNA-protein complexes from lanes 1–6 of **Figure 3B**. Lanes 1′–6′: natural complexes of lanes 1–6 of **Figure 3B**. M, DNA marker. All specific bands ranged from 200 to 1000 bp. D. Endotoxin removed peptides stimulates macrophages to release TNF-α at indicated concentrations. Ana1 macrophages cells were stimulated with HMG peptides at the indicated concentrations, and the culture medium was assayed for TNF-α at 24 h after stimulation. LPS and TgGRA1 were used as controls at the indicated concentrations. Each bar indicates the mean ± SD of three independent experiments. TNF-α secretion induced by TgHMGB1a is significant higher than controls (p<0.01).

To comparative study of the proinflammatory role of TgHMGB1a, peptides (**Figure 3A**) of TgHMGB1a B box (TgH1a B), mouse HMGB1 (mH1), mouse HMGB1 A box (mH1A) and B box (mH1B), and the chimeric protein mH1A+TgH1a B (c (A+B)) were expressed as His-tagged fusion proteins in *E. coli* and purified from soluble form. The purity and molecular weight of the recombinants were examined by SDS-PAGE (**Figure 3B**). Agarose gel electrophoresis assays showed that mH1, TgH1a B, c(A+B), mH1A and mH1Bbound significant amounts of specific DNA fragments between 200 to 1000 bps (**Figure 3C** lanes 1–5), whereas natural recombinant peptides formed complexes with DNA that resulted in retarded bands in the sample loading wells (**Figure 3C** lanes 1′–5′). TgGRA1 did not bind to a specific DNA band as control (**Figure 3C** lanes 6 and 6′), demonstrating that DNA binding is due to the HMG box, but not dependent on the His-tag.

The endotoxin removed recombinants TgHMGB1a 4E stimulates ana1 macrophages to release TNF-α in a dose-dependent manner similar to miceHMGB1 and LPS (positive control) (**Figure 3D**). As the A box of miceHMGB1 usually to be an antagonist of HMGB1, we made a chimeric peptide fused of miceHMGB1 A box and TgHMGB1a B box (c (A+B)) to verify whether or not the A box can also block the TgHMGB1a functions. To test this possibility, we examined the TNF-α secretion in ana1 culture supernatant after c (A+B) stimulation. At higher concentrations (1 and 10 µg/mL), c(A+B) induced TNF-α release significantly less than TgHMGB1a 4E and miceHMGB1 but still higher than the A box, GRA1 and even the B box, demonstrating that miceHMGB1 A box can also be used as an antagonist of TgHMGB1a. To determine whether it is the TgHMG protein alone or in complex with DNA that activates TNF secretion, protein kinase (PK) and DNase I were used to digest the recombinant TgHMGB1a B box respectively, and then we examined the TNF-α levels in the supernatant after stimulated by the treated products. The products after digested by PK almost completely lost the function of induce to TNF-α release, while a slight loss of TNF-α secretion after treat with DNase I. All the results indicated that TgHMGB1a is able to stimulate the TNF-α release in the ana1 macrophages, and which partially but not completely dependents on bound DNA.

### TgHMGB1a specifically binds to cruciform DNA

**Figure 4:**
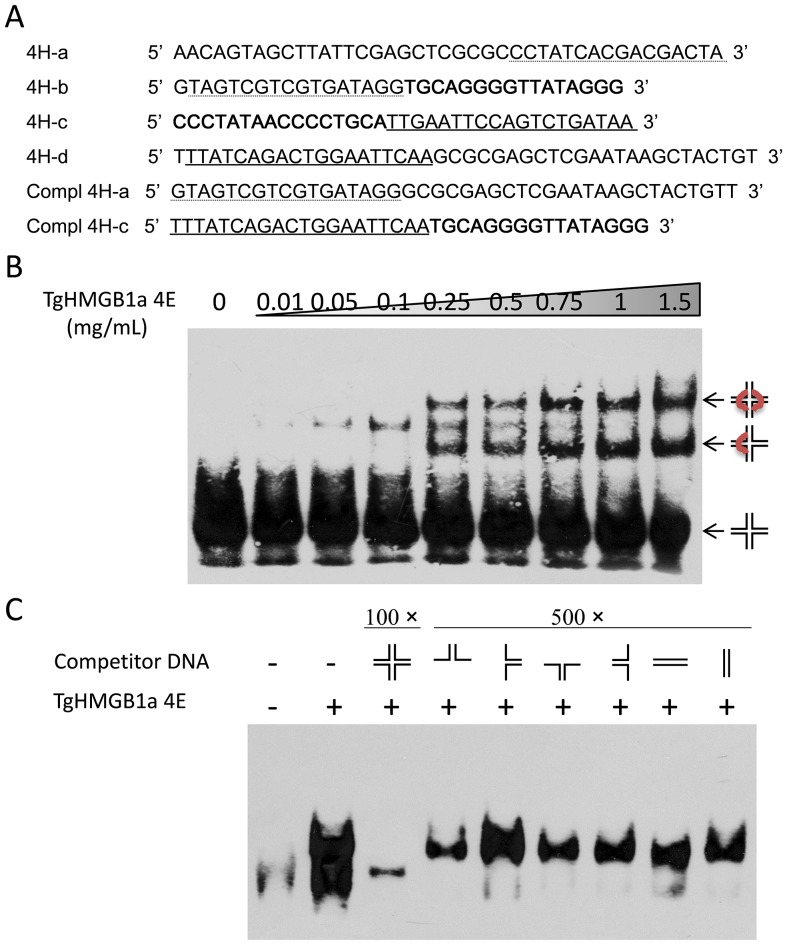
TgHMGB1a binds to cruciform DNA. A. Oligonucleotides used to form the different synthetic DNA structures. Identical text formats (italic, bold, dotted/solid underlines) represent complementary sequences. All of the primers were labeled using EMSA Probe Biotin Labeling Kit as described in methods. Cruciform 4H structures were obtained by annealing equal amounts of 4H-a, -b, -c and -d primers; 2H hairpin-like structures, by annealing of 4H-a and -b, -b and -c, -c and -d, -d and -a, respectively; double stranded (ds) linear DNA structures were obtained by annealing of 4H-a or 4H-c with its fully complementary sequence (compl 4H-a and compl 4H-c). B. EMSA with cruciform DNA (4H) and recombinant TgHMGB1a 4E. Increasing concentrations of TgHMGB1a 4E protein (0.01–1.5 mg/mL) were incubated with biotinlabeled 4H DNA for 30 min at 25°C. DNA–protein complexes formed were resolved on 6% non-denaturing polyacrylamide gel and transferred to nylon membrane and then detected by Chemiluminescent EMSA Kit. Lane 1, free labeled 4H; lanes 2–8, 4H DNA–protein complexes formed with increasing concentrations of TgHMGB1a 4E. C. Competition EMSA experiments were performed with different cold DNA structures. After incubation of TgHMGB1a 4E (1 mg/mL) with biotinlabeled 4H, cold complete 4H (in a 100-fold excess), hairpin-like 2H structures (in a 500- fold excess) or ds linear DNA (in a 500-fold excess) were added, and incubated for an additional 20 min. Complex formation was analyzed as in B. Lane 1, free labeled 4H; lane 2, 4H DNA–protein complexes formed with TgHMGB1a 4E without competitor DNA; lane 3, competition assay with 100-fold excess cold 4H; lanes 4–7 competition assay with 500-fold excess cold 2H hairpin-like structures; lanes 8–9, competition assay with 500-fold excess cold linear ds DNA.

DNA–protein complexes can be analyzed by EMSAs with four-way junction (4H) DNA, preferentially bound by HMGB proteins and a synthetic substrate commonly used to study proteins involved in recognizing and resolving Holliday-type junctions formed during in vivo genetic recombination events (**Figure 4A**). At least two different 4H DNA–TgHMGB1a 4E complexes formed with increasing of the protein concentration, resulting in two discrete retarded bands (**Figure 4B**). These two bands presumably correspond to complexes formed upon binding of one or more TgHMGB1a 4E molecules per 4H DNA. Binding competition with 2H “hairpin like” structures (**Figure 4A and C**, lanes 4–7) demonstrated that TgHMGB1a 4E preferentially binds to complete 4H DNA, since 500-fold excess of these 2H structures does not affect the 4H DNA–TgHMGB1a 4E complex migration. Simultaneously, competition experiments with linear dsDNA derived from the 4H arms (**Figure 4A**) indicated that TgHMGB1a 4E preferentially binds to the DNA cross-over of the cruciform DNA, since 500-fold excess of these duplex DNA molecules were not able to inhibit the formation of the cruciform DNA–TgHMGB1a 4E complexes. As expected, the retarded bands almost disappeared when 100-fold cold 4H was added (**Figure 4C**, lane 3), suggested that there is competition between labeled 4H DNA and excess cold 4H DNA. Purified TgGRA1 was used in similar EMSAs, but it didn't show mobility shift to the cruciform DNA, demonstrating that binding of TgHMGB1a 4E protein is specific and independent of the His-tag fusion (**Figure S7**).

### Disruption of TgHMGB1a did not cause obvious phenotype changes

**Figure 5:**
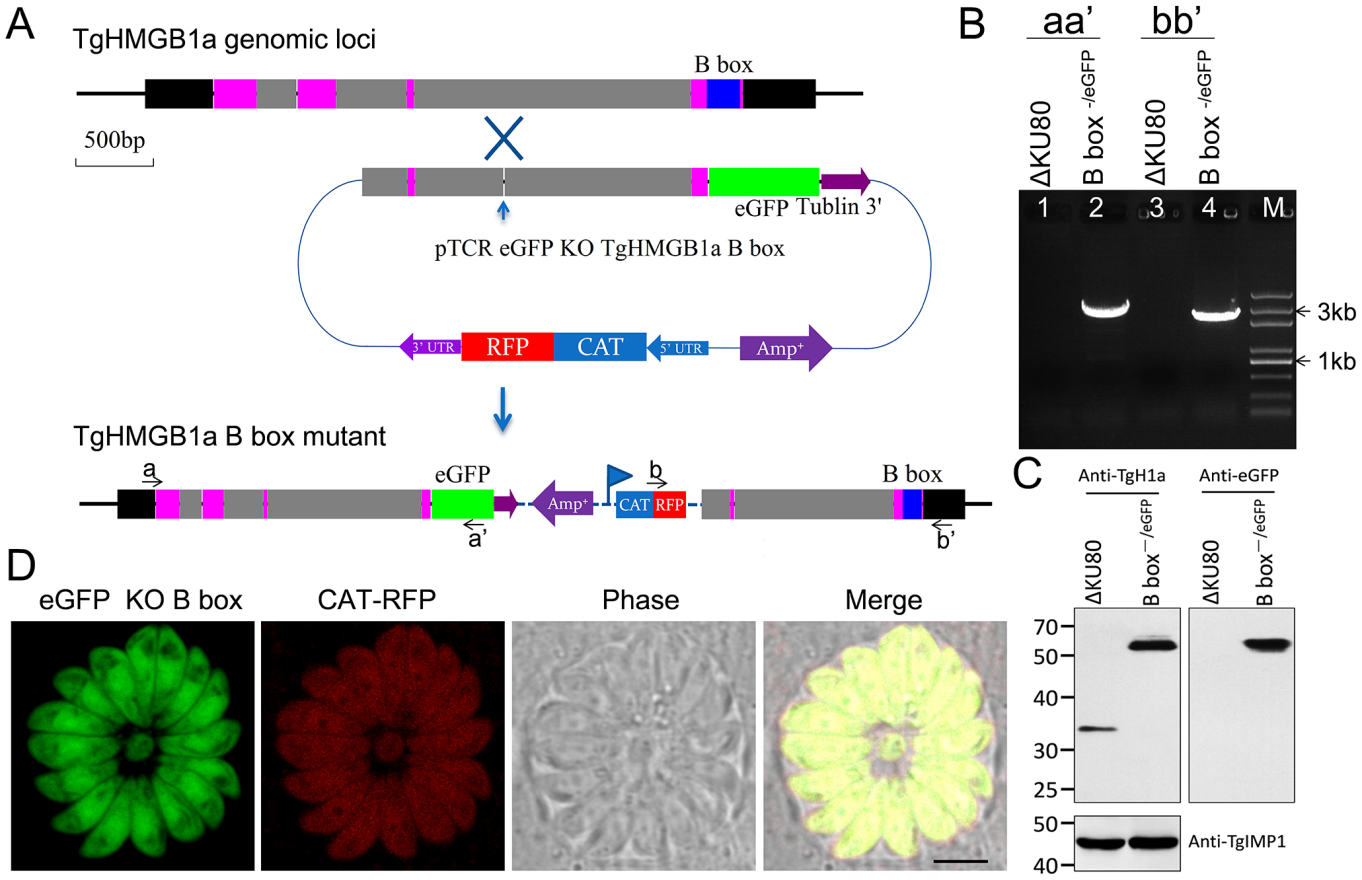
Generation of a TgHMGB1a B box mutant strain. A. Schematic of the experimental design. TgHMGB1a is encoded by 4 exons, and the B box is located in the fourth exon (shown for the TgHMGB1a genomic locus). An “O-type” knock-in/knock-out vector (pTCR eGFP KO TgHMGB1a B box) was constructed to target and replace the B box domain. The vector included an approximately 2200 bp homologous arm upstream of the TgHMGB1a B box, and a monoclonal site of restriction endonuclease (*Bsi*WI) throughout the whole vector sequence was not only needed, but also necessary in the homologous arm, and it is was best ranged around the middle of homologous frame [55]. If the targeted gene was successfully disrupted, eGFP should replace the TgHMGB1a B box domain and create a fusion protein of TgHMGB1a B box^−/eGFP^ expressed under the control of its endogenous promoter. CAT-RFP was used as a selection marker. B. Genomic PCR analysis of the ΔKU80-TgHMGB1a B box^−/eGFP^ strain. The position of the primers and the expected sizes are shown in panel A. C. Western blot performed with anti-TgHMGB1a 4E antibodies on total extracts from RHΔKU80 and ΔKU80-TgHMGB1a B box^−/eGFP^ strains. TgIMP1 was used as an internal control. D. Observation of the TgHMGB1a B box^−/eGFP^ and CAT-RFP strains by fluorescent confocal microscopy, and the result indicated that B box mutant TgHMGB1a was dispersed throughout the parasite cells. Scale bar, 3 µm.

To further characterize the biological role of TgHMGB1a, the B box of TgHMGB1a was targeted and replaced by eGFP in the RHΔKU80 strain using homologous recombination (**Figure 5A**). PCR was used to confirm both replacement of the B box and the insertion of a CAT-RFP cassette in the parasite genome (**Figure 5B**). Replacement of the B box of TgHMGB1a by eGFP was also confirmed using western blotting (**Figure 5C**). We also attempted to generate a complete knockout strain of TgHMGB1a, yet so far have been unsuccessful. Confocal microscopy showed that eGFP was distributed throughout parasite cells, but was not concentrated into the nucleus like integral TgHMGB1a (**Figure 5D**). Nevertheless, the absence of TgHMGB1a B box did not affect parasite growth in vitro, as TgHMGB1a B box^−/eGFP^ parasites formed an equivalent number and size of plaques (**Figure S8A**) and had a replication rate similar to the RHΔKU80 strain (**Figure S8B**). These results suggested that the function of TgHMGB1a may be at least partially redundant, and other homologous proteins may play overlapping roles in parasites.

### Overexpression of TgHMGB1a affects intracellular parasite replication

**Figure 6:**
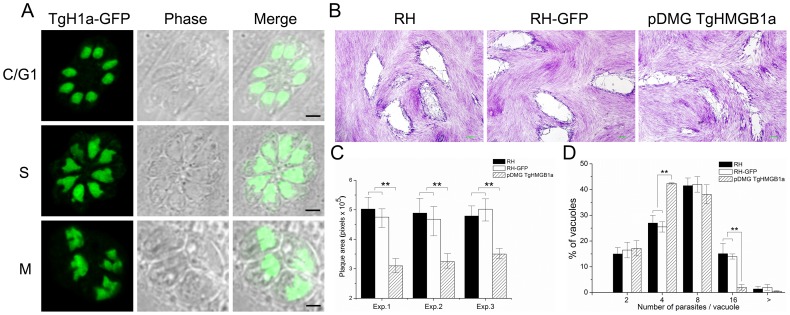
Overexpression of TgHMGB1a affects intracellular parasite replication. A. Transgenic TgHMGB1a localized to the nucleus, similar to endogenous TgHMGB1a. Plasmids of pDMG TgHMGB1a (**Figure S9A**) were introduced into the RH strain to overexpress TgHMGB1a. Through screening, clones were obtained in which the TgHMGB1a-GFP was expressed and concentrated in the nucleus throughout the entire cell cycle (cytokinesis, C; gap phase, G1; DNA synthesis, S; and mitosis, M), which was identical to endogenous TgHMGB1a, as shown by IFA (**Figure 2E**). Western blot were applied to confirm the overexpression of TgHMGB1a (**Figure S9B**). B. Plaque assays for TgHMGB1a overexpression parasites compared to its parental strains. Infected HFF-monolayers were fixed and stained (see Materials and methods). Scale bar, 0.2 mm. C. Plaque assays were carried out three times independently, the plaque area was quantified from three independent experiments. At least 30 plaques were quantified per strain in every experiment. Each bar indicates the mean ± SD; “exp.” indicated the each experiment. D. An intracellular growth rate assay was carried out through counting the number of parasites per vacuole. Parasites of the TgHMGB1a overexpressed and parental strains (RH and RH-GFP) were allowed to invade and divide for 24 hours. IFA was performed with TgSAG1 abs and the numbers of parasites per vacuole were counted. At least 150 vacuoles were examined per strain. Each bar indicates the mean ± SD from three independent experiments; *n*≥150 vacuoles. Statistical significance was determined using Student's *t*-test (**P<0.05).

To make sure that a position effect of the transformation was not involved, we duplicated the experiments with parasite clones isolated from two independent pDMG-TgHMGB1a (**Figure S9A**) transfections. Meanwhile, to determine the phenotype changes were due to the HMG domain of TgHMGB1a specifically, a strain overexpressing a construct lacking the HMG domain was also generated as control. TgHMGB1a overexpression was confirmed by western blotting (**Figure S9B**) and more importantly, exogenous TgHMGB1a-GFP was concentrated in the nucleus throughout the development of intracellular *T. gondii*, which colocalized with endogenous TgHMGB1a (**Figure 6A**), and B box lacked TgHMGB1a hasn't concentrated in nucleus but likely dispersed in cytoplasm (**Figure S9C**), which suggested the B box domain might effects on the localization of TgHMGB1a. Plaque assays showed that the RH and RH-GFP strains did not form significantly different plaques sizes, but the transgenic parasites generated plaques that were smaller than the ones generated by its parental strain (**Figure 6B**), and almost there were no significant difference between the TgHMGB1a^−B box^ parasites and RH or RH-GFP strains (**Figure S9D**). In another words, TgHMGB1a overexpression inhibited the formation of large plaques. Furthermore, it's most due to the HMG domain of TgHMGB1a. Quantification of plaque areas suggested that the size of plaques formed by RH and RH-GFP strains was almost two-fold greater than the ones formed by the transgenic parasites (**Figure 6C**). Furthermore, comparison of the replication rates showed a significant difference in the distribution of parasites per vacuole between the transgenic parasites and its parental strain (**Figure 6D**); the transgenic parasites more often contained 4 and 8 tachyzoites vacuoles than its parental strain, while the parental strain more often contained 8 and 16 parasites vacuoles. Thus, these data suggested that overexpression of TgHMGB1a led to slower growth during intracellular replication in vitro.

### TgHMGB1a involved in the regulation of gene transcription

**Figure 7:**
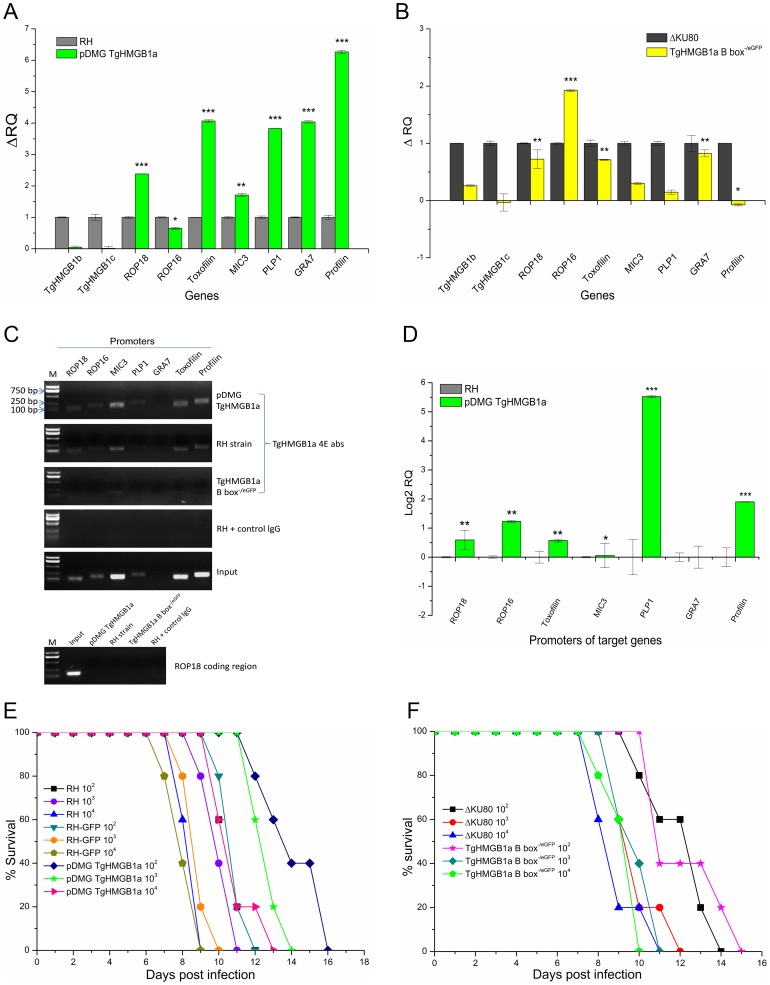
TgHMGB1a involves to gene transcription regulatory and overexpress but not disrupt TgHMGB1a can delayed the death of mice. A and B. Quantitative RT-PCR was used to analyze the transcription levels of the indicated genes in TgHMGB1a overexpression (A) and B box-deficient (B) parasites compared with their parental strains. Each bar indicates the relative quantity (RQ) ± SD. RQs of the transgenic parasites were calibrated using their parental strains (i.e., ΔRQ_transgenic parasite_ = RQ_transgenic parasite_ – RQ_parental strain_( = 1)). Data presented are representative of three independent experiments, each done in triplicate. Statistical significance was determined using Student's *t*-test (*P<0.1, **P<0.05, ***P<0.005). C and D. Analysis of TgHMGB1a binds to promoters of indicated genes through chromatin immunoprecipitation (ChIP). C. Regular PCR was performed on the ChIP DNAs from the TgHMGB1a overexpress and RH strains using the promoter-specific primers of indicated genes, normal mice sera, input DNA and TgHMGB1a B box^−/eGFP^ strain were used as ChIP controls, ROP18 coding region was also tested as negative control. PCR products were run on 1.5% agarose gels. D. Quantitative real-time PCR was carried out and the pull-down promoters in RH and TgHMGB1a overexpress strains were normalized against the corresponding input DNA, the promoters level in TgHMGB1a overexpress parasites were represented as log2 functions of relative ratios to RH strain. Data presented are representative of three independent experiments, each done in triplicate. Analysis was carried out using Student's t-test. *P<0.1, **P<0.01, ***P<0.001. E and F. Mouse survival curves for parental and TgHMGB1a transgenic lines. Balb/c mice were separately injected i.p. with the indicated parasites and doses. Mean values shown per group (n = 5), representative of 3 experiments with similar outcomes. Mice infected with the TgHMGB1a overexpression showed a significantly delayed time to death (3 to 5 days) in a low doses (10^2^ and 10^3^) infection compared to its parental RH or RH-GFP strains (*P*<0.0001 was considered as statistically significant difference, see the **Data S1**).

Quantitative RT-PCR analysis of 1×10^7^ transgenic and their parental parasites were conducted to determine the potential involvement of TgHMGB1a in transcriptional regulation. Our quantitative RT-PCR analysis indicated that ROP18, toxofilin, PLP, MIC3, profilin, and GRA7, but not ROP16, were up regulated at least 1.5-fold in TgHMGB1a overexpression parasites (**Figure 7A**), yet there were no obvious changes observed in the TgHMGB1a B box^−/eGFP^ strain. However, a dramatic increase of ROP16 expression was detected in the TgHMGB1a B box^−/eGFP^ strain (**Figure 7B**), even though profilin expression was reduced. Expression of the TgHMGB1a homologues, TgHMGB1b and TgHMGB1c, were also measured. TgHMGB1b and TgHMGB1c were almost undetectable and similarly expressed in transgenic and parental strains, while TgHMGB1a was overexpressed (**Figure 7A**). However, in the TgHMGB1a B box^−/eGFP^ parasites, TgHMGB1b showed a significant increase, whereas TgHMGB1c showed a nonsignificant trend towards reduced expression (**Figure 7B**). Furthermore, ChIP-qPCR analysis showed that promoter levels of PLP1, profilin, ROP16, ROP18 and Toxofilin in the pull down of TgHMGB1a overexpress strain are significant higher than RH strain, although MIC3 and GRA7 are not obvious (**Figure 7C and D**). Meanwhile, there are almost no signals in TgHMGB1a B box^−/eGFP^ parasites and in the negative controls (**Figure 7C**). Moreover, we also tested the coding regions through the regular and real-time PCR, and results showed no significant difference between the wild type and TgHMGB1a overexpress strain (**Figure 7C** **bottom panel** and data not shown). These results suggested that TgHMGB1a overexpress enhanced the promoters binding ability, that is generally consistent with the transcription assays of these indicated genes (**Figure 7A**), demonstrating that TgHMGB1a plays role in regulating gene expression, and which is most like an activator of transcription and its roles might be somewhat redundant among HMG family members.

### Overexpress but not disrupt TgHMGB1a *T.gondii* delayed the death of mice

To evaluate the contribution of TgHMGB1a to parasite virulence, we measured the survival of mice infected with the transgenic *T. gondii* strains or their parental strains. Consistent with the in vitro assays, the survival of Balb/c mice infected with TgHMGB1a B box^−/eGFP^ or RHΔKU80 strains were not significantly different (**Figure 7F**). However, mice infected with the TgHMGB1a overexpression showed a significantly delayed time to death (3 to 5 days) in a low doses (10^2^ and 10^3^) infection compared to its parental RH or RH-GFP strains (*P*<0.0001 was considered as statistically significant difference, see the **Data S1**), while the RH and RH-GFP strains showed almost equivalent virulence (**Figure 7E**). Collectively, these results established that the expression of TgHMGB1a is involved in parasitic virulence, however, it function may be partially redundant.

## Discussion

Homologous HMG proteins have been reported in many parasites, and almost all of these proteins share the characteristics of DNA binding ability and nuclear localization. However, the endogenous functions of these proteins are poorly understood in parasites. Previous studies indicated that most parasite HMGB1s are expressed throughout the life cycle of a parasite. In *S. mansoni*, HMGB1 appears to be a stage-specific protein that is abundantly expressed in the skin-stage schistosomula, adult female and egg stages of the parasite, whereas its expression is low or absent in the adult male and cercarial stages [16]. In *E. histolytica*, overexpression of EhHMGB1 caused the modulation of 33 transcripts, including four virulence factors that are involved in various cellular functions [21]. In *P. falciparum*, two HMGB factors were identified, and in vitro analyses showed that they were able to interact with distorted DNA structures and bend linear DNA with different affinities and to play roles in transcriptional regulation of *Plasmodium* development in erythrocytes [19], [20]. In this study, many members of the HMGB1 family have been predicted in *T. gondii* (**Figure S1 and Table S6**). We cloned and characterized *T. gondii* HMGB proteins, and our results suggested that structure and functions of TgHMGB1a are similar to HMGB1 family proteins in their DNA binding ability and nuclear localization. Our findings confirmed that TgHMGB1a is expressed in *T. gondii*, and which were conserved in three genotypes (**Table S1**). However, region of TGGT1_053220 (ToxoDB ver.9.0) has been renamed two independent genes (TGGT1_210412 and TGGT1_210408 in ToxoDB ver.11), the western blot showed a protein with molecular weight (about 33.9 kDa) in line with the TGGT1_053220. So our results suggested that TGGT1_210408 may be the last exon of TGGT1_210412, at least, there is a combined transcript of this two fragments (TGGT1_210408 and TGGT1_219828 in ToxoDB ver.11) in RH strain.

Both our sequence and structure analyses converged, indicating that only one *T. gondii* HMG box domain closely resembles the box B of mammalian HMGB (**Figure 1**). For human HMGB1, it was reported that structure-specific binding to cruciform DNA was mediated by box A and that box B, flanked by a basic region, displayed a marked DNA recognition activity [38], [43]. In this study, we verified that TgHMGB1a B box can bind the DNA fragments of *E. coli* (**Figure 3**), however, the differences of the bound DNA might have resulted from the varying degrees of sonication. Nevertheless, many HMGB1 proteins in other organisms, for example *Plasmodium* [19], showed no specific binding ability to the DNA that interacted specifically with typical SOX and SRY transcription factors (TFs) in an EMSA. We approved the TgHMGB1a B box specifically binds the cruciform DNA in a high affinity (Fig 4) like the *T.cruzi* HMGB proteins [14], which may be valuable to understand the interaction between TgHMGB1s and DNA. Like the mammals HMGB1, parasites HMGB1 in general characterized by DNA binding ability and proinflammatory responses [20]. Comparison of the ability of induce to TNF-α release by the indicated recombinants (**Figure 3D**) showed that TgHMGB1a is able to stimulate the macrophages to release TNF-α as miceHMGB1, and the results of PK and DNase I treatments suggested that TgHMGB1a induced to TNF-α secretion is partially but not completely dependent on the bound DNA, however, it's more importantly investigate to reveal how does a nuclear factors contact to host immune system, and actually we haven't detected TgHMGB1a secretion at least in culture supernatant of type I parasites (Data not shown). These results suggest that TgHMGB1s may be related to host inflammatory immune responses in *T.gondii* infection.

In this study, two transgenic parasite strains were generated to examine the roles of TgHMGB1a in *T. gondii*. One strain is the TgHMGB1a B box^−/eGFP^ mutant, and the other is overexpressed TgHMGB1a. We measured the intracellular growth ability of the transgenic parasites using plaque assay, and our results showed slower growth of the TgHMGB1a overexpression strain (**Figure 6**), but no significant changes in the TgHMGB1a B box^−/eGFP^ mutant compared with its parental strains (**Figure S8**). We also demonstrated that overexpressing TgHMGB1a or deleting the TgHMGB1a B box modulated the expression of many virulence factor genes (**Figure 7A and B**). ROP18 and ROP16 were found to be the most important virulence factors in *T. gondii*, parasites that express the Type I ROP18 allele display rapid cell cycle times in cell culture and transfer of the Type I-ROP18 allele into avirulent strains speeds their growth (but not their ability to invade) and dramatically enhances virulence [44], nevertheless, how does ROP18 regulates the parasite growth rate is unknown. ROP18 might influences the parasite cell cycle mechanisms [45] but not be the sole determinant for parasite growth, for which is a so complex process involved a great many genes regulatory and pathways. So, upregulation of ROP18 is not necessarily leads to a faster grow rate, and by contrast, the slower growth rate showed in TgHMGB1a overexpression parasites indicated that TgHMGB1a may be involved to cell cycle regulations. On the other hand, types I and III ROP16, but not type II ROP16, can maintain constitutive activation of STAT3 and STAT6, thereby inhibiting pro-inflammatory responses in macrophages (reviewed by [27]) and conferring a survival advantage to the parasite. In the TgHMGB1a B box^−/eGFP^ parasites, ROP16 was significantly upregulated and may have partially neutralized any negative affect by downregulating other genes in vivo, for instance, the recognition of profilin through the Toll like receptor 11 (TLR-11), that mediates production of IL-12 and triggers a strong Th1-type response to against *T.gondii* infection [46]. The TgHMGB1a overexpression strain caused delayed death in Balb/c mice for that the slower growth rate might attenuate parasitic virulence [30], [47], but there were no meaningful changes in the TgHMGB1a B box^−/eGFP^ mutant compared with its parental strains, in agreement with the results obtained in vitro.

Intuitively, all of these results suggested that TgHMGB1a participates in these processes as a negative regulator, which is not consistent with the typical functions of HMGB1 proteins. However, we cannot conclude that TgHMGB1a is a transcriptional repressor in *T. gondii*; at best, we can only show that it is a repressor of parasite proliferation. In fact, in eukaryotes, the association of HMGBs with chromatin is highly dynamic, and the proteins affect the chromatin fiber as architectural factors by transient interactions with nucleosomes [2], [48]. It has been proposed that histones and HMGB1s have opposite effects; histone H1 molecules are considered to be general repressors of transcription, whereas HMGBs are often viewed as transcriptional activators [49]. While the linker histones and HMGBs exhibit direct competition in binding to such structures as four-way junction DNA, whether they compete for binding to the nucleosome has not been investigated. The possibility for either opposite or synergistic effects on gene regulation must be considered at this point [49], and future investigations will be necessary to increase our knowledge of the interactions between HMGB1 and histones in *T. gondii*. However, there is no Histone H1 in *T. gondii* but in addition to having a single copy of canonical histones (H2A, H2B, H3 and H4), *T. gondii* encodes five variant histones (centromeric H3 (CenH3), H3.3, H2A. X, H2A. Z, and the parasite-specific H2Bv [50]. Such studies might provide exciting new insights to advance understanding of the transcription-related functions of TgHMGBs.

More direct and very important evidence for the involvement of TgHMGB1a in transcription regulation was generated by quantitative RT-PCR studies, which indicated that the transcription levels of many genes were elevated in the TgHMGB1a overexpression parasites, but were not significantly reduced in the TgHMGB1a B box-deficient strain. Nevertheless, it's more like that TgHMGB1a is negative regulated for ROP16 but promotes transcription of profilin (**Figure 7A and B**), however, it needs to be further investigated. Furthermore, promoter binding assays with ChIP-qPCR analysis showed that TgHMGB1a prefers bind to promoter regions (**Figure 7C and D**) and maybe in non-gene-specific promoter manner (most likely as an architectural factor of the transcription complex). Collectively, we suggest that TgHMGB1a plays an activator role in gene transcription regulation in *T. gondii*, as in mammals, and we supposed that the replication of *T. gondii* was controlled by cell cycle regulatory genes [47], [51], which were upregulated in the TgHMGB1a overexpression strain, enhancing the role of inhibition for replication, and then the parasites showed slower growth compare with their parental strain. The mRNA levels of the other HMGB1s in *T. gondii*, TgHMGB1b and TgHMGB1c, were also measured, and only TgHMGB1b showed a slight increase and TgHMGB1c declined. Therefore, it remains unclear whether there is a feedback mechanism involving HMGB1 proteins that affects the synthesis of these genes, with the view of their functions may be partially redundant and compensated for when one is deficient in parasite. However, polymorphisms in HMGB1 genes may support partial redundancy among HMGB1 proteins.

The DNA-related functions of TgHMGB1a are consistent with its nuclear location (**Figure 2**). Interestingly, no canonical NLS was found in TgHMGB1a by bioinformatic predictions, and there were no signal sites identified in the B box, even though B box-deficient TgHMGB1a (**Figure 5** **and Fig S9C**) did not concentrate in the nucleus but dispersed throughout the parasite cells (**Figure 6D**). In other species, the acetylation status of HMGB proteins can alter both their DNA-binding properties and their subcellular localizations [52], [53]; N-myristoylation and palmitoylation sites have also been shown to contribute to protein localization [54]. Although one N-myristoylation (100–105 aa) and two tyrosine kinase phosphorylation (282–290 and 289–297 aa) sites were identified in TgHMGB1a, further studies will be needed to determine whether PTMs of TgHMGBs affect the localization of these proteins in *T. gondii*. Investigations of tyrosine kinase phosphorylation sites will be especially interesting, because they have been studied little in this context. Through comparison of TgHMGB1a localization between the intracellular and extracellular, we found an interesting phenomenon that TgHMGB1a showed a translocation after the parasites released to extracellular (**Figure S5 and S6**). Usually, the transcription of most metabolic genes should be reduced when the parasites are outside of the cell, so it may be a protection mechanism of parasites for TgHMGB1s translocated out of the nuclear perhaps because of the non-specific transcription activation of TgHMGB1s.

In conclusion, all of our computational analysis and experimental results were concordant suggested that the TgHMGB1s were substantially similar to multicellular HMGB proteins in both structure and functions. TgHMGB1a was implicated in transcriptional regulation and most likely acts as an activator of many virulence factors. Additional work will be needed to understand the mechanisms of TgHMGB1a and the HMG family proteins in *T. gondii*, both as nuclear factors and also proinflammatory mediators. To our knowledge, this is the first report of a role for an HMGB1 protein in transcriptional regulation of *T. gondii* genes that are involved in intracellular parasite replication and maybe affect parasite virulence in mice.

## Supporting Information

Figure S1 **Phylogenetic analysis of HMGBs in various organisms.** A. Phylogenetic analysis based on HMGB proteins from various organisms. HMGB proteins from Apicomplexa and other parasites, humans, mammals, D. rerio, A. thaliana and S. cerevisiae were analyzed based on either the neighbor-joining (NJ) method of distance analysis or the maximum likelihood (ML) method. The bootstrap consensus tree inferred from 1000 replicates is thought to represent the evolutionary history of the taxa analyzed. The tree includes TgHMGB1a (light green circle), TgHMGB1b (light blue circle), TgHMGB1c (light cyan circle) and homologues found in other eukaryotes. Bootstrap values (>50%) from 1000 resamplings are indicated prior to the branch points of the tree. Only bootstrap values >95 were considered to be significant and allowed to form clustered sequences (colored branches). Protein numbers were given according to the EuPathDB and NCBI websites. Also, see Table S1 for the multiple sequence alignment used to compute the phylogenetic trees. B. Phylogenetic analysis based on the CDS of HMGB1s from various organisms. The coding sequences of HMGB proteins from Apicomplexa and other parasites, human, mammals, D. rerio, A. thaliana and S. cerevisiae were analyzed based on neighbor-joining (NJ) using MEGA 5.22 (1000 bootstrap replicates were performed). The results are consistent with phylogenetic analysis based on the amino acid sequences. TgHMGB1a, b and c are branched three clusters and all they are the closest relatives to HMGB1 of Homo sapiens. Abbreviations are as follows: Hs, Homo sapiens; Xl, Xenopus laevis; Dr, Danio rerio; At, Arabidopsis thaliana; Eh, Entamoeba histolytica; Sc, Saccharomyces cerevisiae; Pf, Plasmodium falciparum; Nc, Neospora caninum; Tg, Toxoplasma gondii; Et, Eimeria tenella; Lm, Leishmania major; Tc, Trypanosoma cruzi; Tb, Trypanosoma brucei; and Sm, Schistosoma mansoni.
(TIF)

Figure S2 **HMG box contained proteins in mice and** ***T. gondii*** **.** Mouse HMGB1 has 2 HMG boxes, whereas all TgHMGB1s but not TGGT_203950have only one HMG box. TGGT1_053220 (ToxoDB ver.9,0) was renamed as TGGT1_210412 in ToxoDB ver.11, and the last exon of TGGT1_053220 has been named TGGT1_210408 in ToxoDB ver.11, however, our results suggested that there might be a combined transcript of this two fragments (TGGT1_210408 and TGGT1_219828), at least, that is TgHMGB1a named in present study. Same to TGGT1_053220, TGGT1_030840 was also renamed, and N-terminal region has been predicted as cyclin-dependent protein serine/threonine kinase regulator subunit protein (TGGT1_219832). The others, such as TGGT_203950 (three HMG box, showed only two box for the long sequence structure) and TGGT_217500 (N-terminal HMG box) should be not classified into HMGB proteins for the low structure similarity and long genetic distance (Figure S1).
(TIF)

Figure S3 **Sequence-based bioinformatic predictions of TgHMGB1a.** A. TgHMGB1a is predicted to lack a signal peptide sequence. B. The four hydrophobic transmembrane domains at the N-terminus are shown. C. Prediction of post-translational modifications of TgHMGB1a. Sites for protein kinase C phosphorylation (14–16 aa), N-glycosylation (83–86 aa), N-myristoylation (100–105 aa), amidation (141–144 aa), casein kinase II phosphorylation (185–188 aa) and tyrosine kinase phosphorylation (282–290 and 289–297 aa) sites were where indicated. Also, proprotein convertase sites were found at the C-terminal.
(TIF)

Figure S4 **TgHMGB1a constantly express in the intracellular parasites but dramatically decreased in extracellular parasites.** The intracellular parasites and freshly released parasites were collected to lysed using RIPA (strong) buffer added cooktail proteinase inhibitor for 30 min on ice, and then analyzed by western blot with anti-TgHMGB1a 4E sera. Meanwhile, freshly released tachyzoites (extracellular 0 h) were incubated additional 0.5, 2 and 6 h in 10% FBS DMEM at 37°C, then centrifuged (800 g, 8 min) and collected the pellets to analyze together with the intracellular parasites. TgActin abs was used to normalize the quantity of per samples.
(TIF)

Figure S5 **Localization of TgHMGB1a in the intracellular stage.** Top panels: TgHMGB1a always concentrated in the parasites nuclear during the course of endodyogeny. Anti-MIC3 antibodies were used as the control (indicated cytoplasm region). Scale bar, 3 µm. Bottom panels: TgHMGB1a is concentrated in the nucleus and less dispersed in the cytoplasm. Anti-SAG1 antibodies were used to label the cytoplasm membrane. Scale bar, 3 µm.
(TIF)

Figure S6 **Localization of TgHMGB1a in the extracellular stage.** Top panels: TgHMGB1a translocates into cytoplasm when the parasites released to extracellular. Anti-MIC3 antibodies were used as the control. Scale bar, 5 µm. Bottom panels: TgHMGB1a translocates into cytoplasm but not enriched in nucleus when the parasites released to extracellular. Anti-SAG1 antibodies were used to label the cytoplasma membrane. Top panel scale bar is 3 µm and bottom panel scale bar is 3 µm.
(TIF)

Figure S7 **EMSAs with cruciform DNA (4H DNA) and recombinant TgHMGB1a 4E protein or recombinant TgGRA1 protein. Increasing concentrations of TgHMGB1a 4E protein (0.1–6 mg/mL) were incubated with 5 nmol of biotinlabeled 4H DNA for 30 min at 25°C, 2 mg/mL TgGRA1 was used as control.** DNA-protein complexes formation were resolved on 6% non-denaturing polyacrylamide gel and transferred to nylon membrane and then detected by Chemiluminescent EMSA Kit. Under identical conditions, only the TgHMGB1a 4E protein can binds cruciform DNA, but not an electrophoretic mobility shift of the 4H probe with TgGRA1 even though in a high concentration.
(TIF)

Figure S8 **Phenotypic analysis of the TgHMGB1a B box-/eGFP mutant strain.** A. Monolayers of HFF were infected with RHΔKU80 or TgHMGB1a B box-/eGFP parasites. Plaques were allowed to form undisturbed and, at 7 days post inoculation, the monolayer was fixed and stained (see Materials and methods). B. An intracellular growth rate assay was carried out after 24 hr of parasite growth. IFA was performed with TgSAG1 abs and the numbers of parasites per vacuole were counted. Each bar indicates the mean ± SD from three independent experiments; n≥150 vacuoles.
(TIF)

Figure S9 **TgHMGB1a overexpression in the RH strain.** A. Schematic of the plasmid transfected into the RH strain for overexpression of TgHMGB1a. TgHMGB1a was fused to GFP, which was under the control of the GRA1 promoter. B. TgHMGB1a overexpression was confirmed by western blotting. As expected, transfected clones showed two TgHMGB1a bands (i.e., one is endogenous TgHMGB1a and the other is TgHMGB1a-GFP). When anti-GFP antibodies were used, only the transfected parasite antigens showed specific binding. As an internal control, polyclonal antibodies against T. gondii immune mapped protein 1 (TgIMP1) were used. C. TgHMGB1a-B box-GFP was not concentrated in the nucleus. The plasmid of pDMG TgHMGB1a-B box were transfected into RH strain, and the monoclone were obtained by FCM sorting. B box deficient TgHMGB1a has not localized in nuclear of parasites as showed by the fused GFP, and it likely dispersed in cytoplasm. D. To analyze whether the phenotypes changes were specific to the HMG domain of TgHMGB1a in the overexpress strains, pDMG TgHMGB1a-B box, pDMG TgHMGB1a transfected clones and their parental strains (RH and RH-GFP) were examined by plaque assays. The results showed that only plaques of pDMG TgHMGB1a transfected clones were smaller than their parental strains, demonstrating the HMG domain is involved to the phenotype changes. These pictures were representative of 3 experiments with similar outcomes. Scale bar, 0.2 mm.
(TIF)

Table S1 **Sequences were used to phylogenetic analysis.**
(DOCX)

Table S2 **Primers used for clone and prokaryotic expression.**
(DOCX)

Table S3 **Primers used for TgHMGB1a overexpress and B box mutation.**
(DOCX)

Table S4 **Primers used for qRT-PCR.**
(DOCX)

Table S5 **Primers of target gene promoters used for ChIP-qPCR.**
(DOCX)

Table S6 **Repertoire of high mobility group box proteins in three genotypes** ***T.gondii*** **.**
(DOCX)

Data S1 **Completed ARRIVE checklist for animal infection experiments.**
(DOCX)
