# Supplementary figures and images for: A Nuclear Factor of High Mobility Group Box Protein in Toxoplasma gondii
Source: PLoS One. 2014 Nov 4;9(11):e111993. doi: 10.1371/journal.pone.0111993 (PMC4219823; doi:10.1371/journal.pone.0111993)

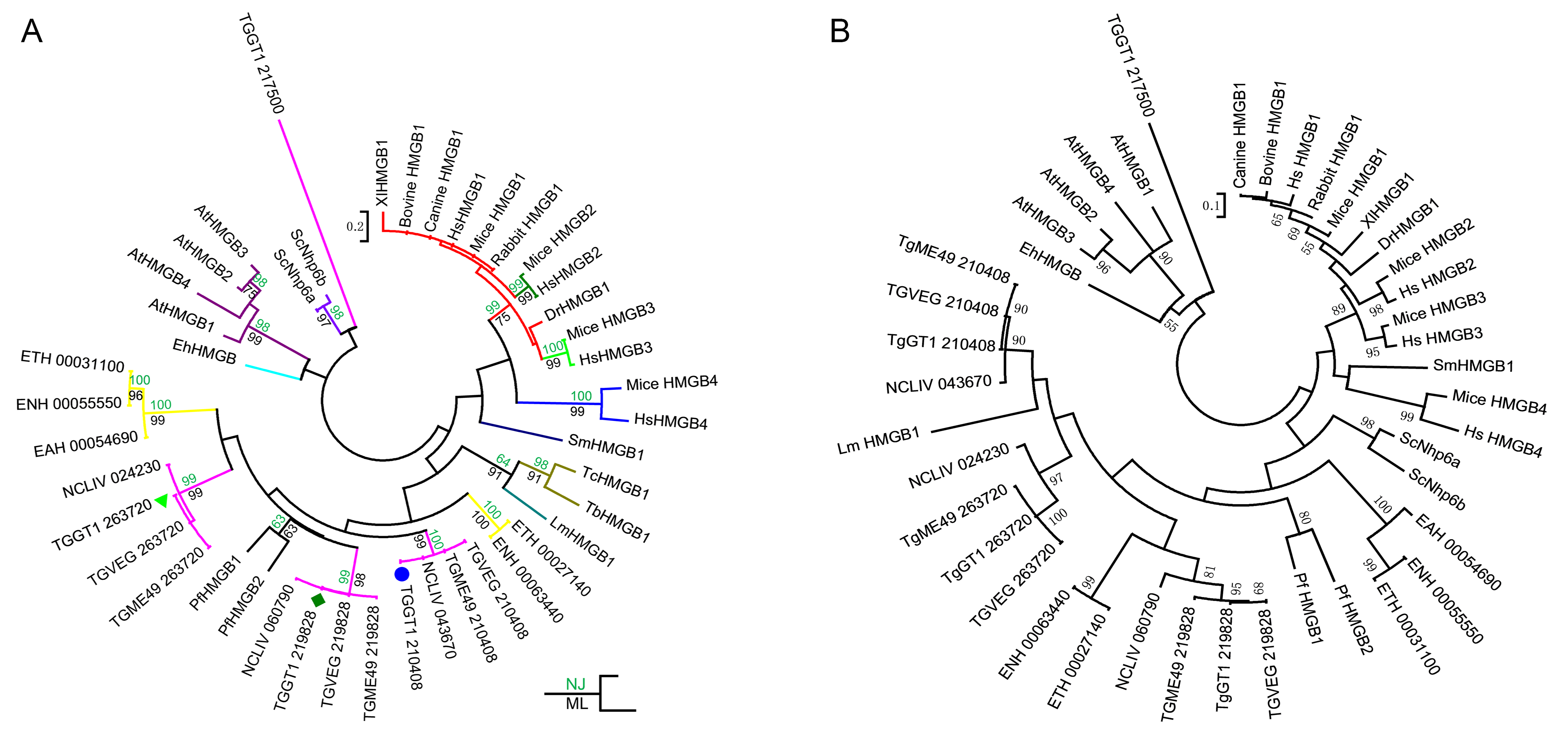

Supplement: Figure S1 — Phylogenetic analysis of HMGBs in various organisms. A. Phylogenetic analysis based on HMGB proteins from various organisms. HMGB proteins from Apicomplexa and other parasites, humans, mammals, D. rerio, A. thaliana and S. cerevisiae were analyzed based on either the neighbor-joining (NJ) method of distance analysis or the maximum likelihood (ML) method. The bootstrap consensus tree inferred from 1000 replicates is thought to represent the evolutionary history of the taxa analyzed. The tree includes TgHMGB1a (light green circle), TgHMGB1b (light blue circle), TgHMGB1c (light cyan circle) and homologues found in other eukaryotes. Bootstrap values (>50%) from 1000 resamplings are indicated prior to the branch points of the tree. Only bootstrap values >95 were considered to be significant and allowed to form clustered sequences (colored branches). Protein numbers were given according to the EuPathDB and NCBI websites. Also, see Table S1 for the multiple sequence alignment used to compute the phylogenetic trees. B. Phylogenetic analysis based on the CDS of HMGB1s from various organisms. The coding sequences of HMGB proteins from Apicomplexa and other parasites, human, mammals, D. rerio, A. thaliana and S. cerevisiae were analyzed based on neighbor-joining (NJ) using MEGA 5.22 (1000 bootstrap replicates were performed). The results are consistent with phylogenetic analysis based on the amino acid sequences. TgHMGB1a, b and c are branched three clusters and all they are the closest relatives to HMGB1 of Homo sapiens. Abbreviations are as follows: Hs, Homo sapiens; Xl, Xenopus laevis; Dr, Danio rerio; At, Arabidopsis thaliana; Eh, Entamoeba histolytica; Sc, Saccharomyces cerevisiae; Pf, Plasmodium falciparum; Nc, Neospora caninum; Tg, Toxoplasma gondii; Et, Eimeria tenella; Lm, Leishmania major; Tc, Trypanosoma cruzi; Tb, Trypanosoma brucei; and Sm, Schistosoma mansoni. (TIF) [file pone.0111993.s001.tif]

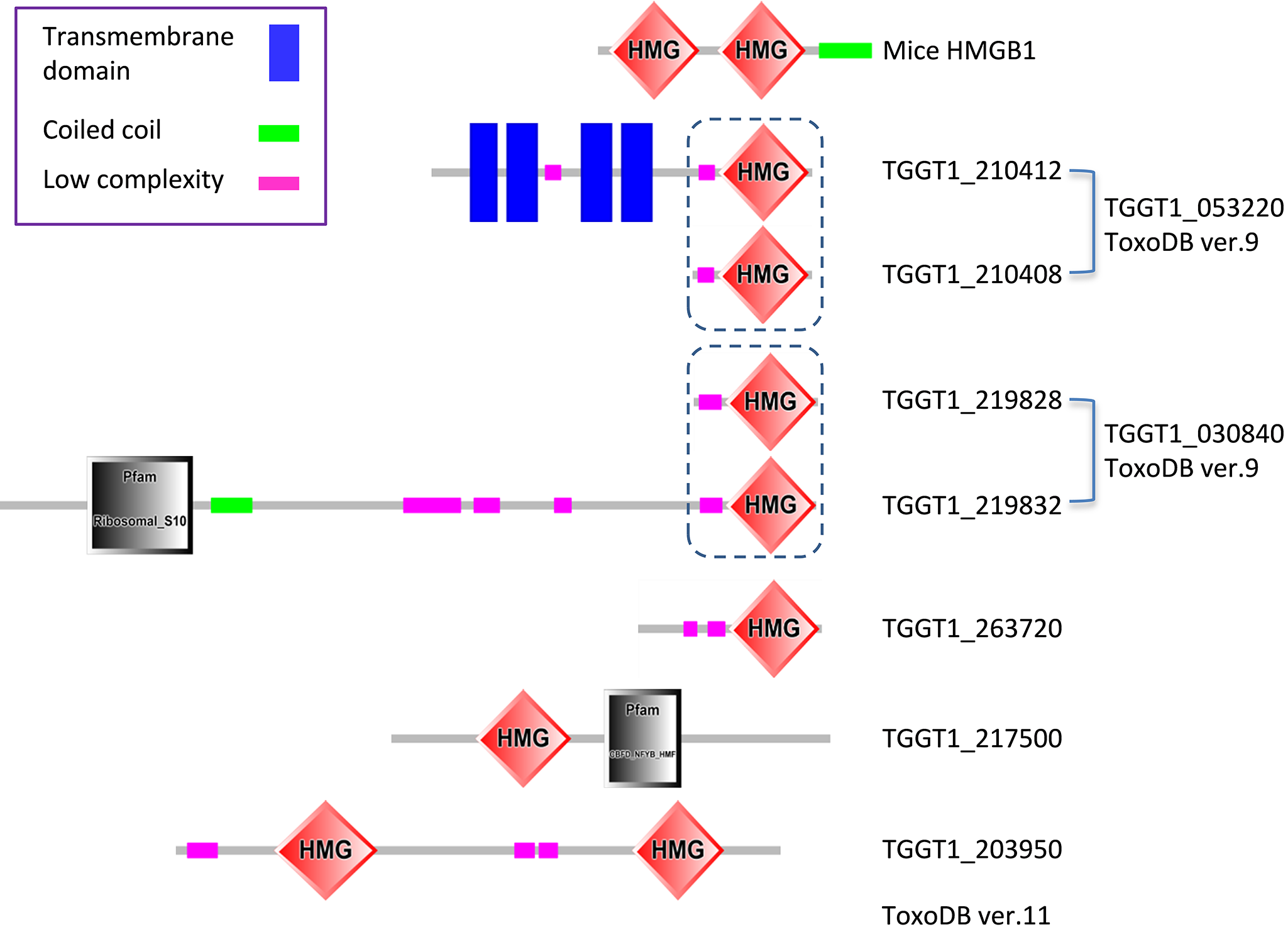

Supplement: Figure S2 — HMG box contained proteins in mice and T. gondii . Mouse HMGB1 has 2 HMG boxes, whereas all TgHMGB1s but not TGGT_203950have only one HMG box. TGGT1_053220 (ToxoDB ver.9,0) was renamed as TGGT1_210412 in ToxoDB ver.11, and the last exon of TGGT1_053220 has been named TGGT1_210408 in ToxoDB ver.11, however, our results suggested that there might be a combined transcript of this two fragments (TGGT1_210408 and TGGT1_219828), at least, that is TgHMGB1a named in present study. Same to TGGT1_053220, TGGT1_030840 was also renamed, and N-terminal region has been predicted as cyclin-dependent protein serine/threonine kinase regulator subunit protein (TGGT1_219832). The others, such as TGGT_203950 (three HMG box, showed only two box for the long sequence structure) and TGGT_217500 (N-terminal HMG box) should be not classified into HMGB proteins for the low structure similarity and long genetic distance (Figure S1). (TIF) [file pone.0111993.s002.tif]

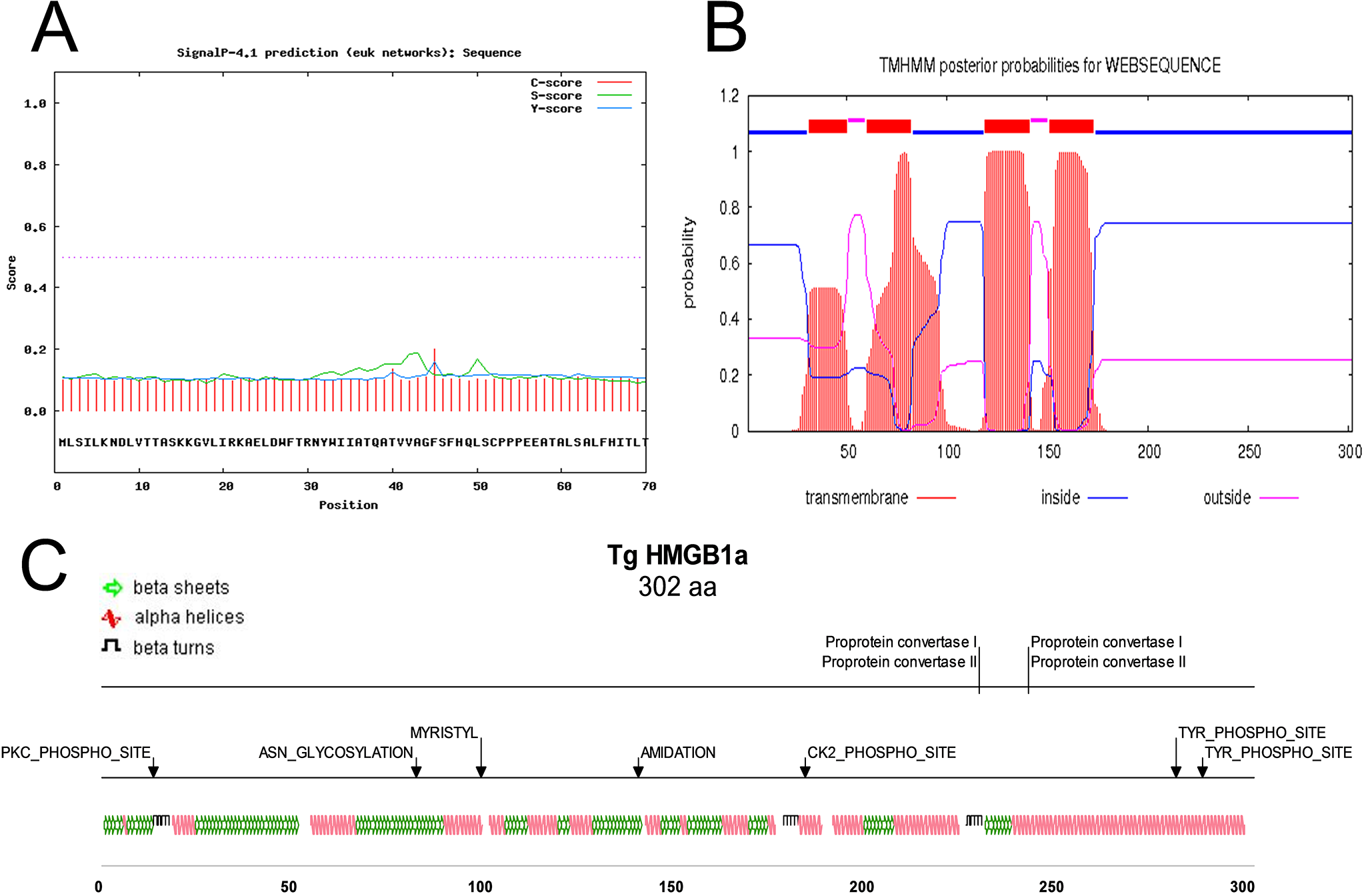

Supplement: Figure S3 — Sequence-based bioinformatic predictions of TgHMGB1a. A. TgHMGB1a is predicted to lack a signal peptide sequence. B. The four hydrophobic transmembrane domains at the N-terminus are shown. C. Prediction of post-translational modifications of TgHMGB1a. Sites for protein kinase C phosphorylation (14–16 aa), N-glycosylation (83–86 aa), N-myristoylation (100–105 aa), amidation (141–144 aa), casein kinase II phosphorylation (185–188 aa) and tyrosine kinase phosphorylation (282–290 and 289–297 aa) sites were where indicated. Also, proprotein convertase sites were found at the C-terminal. (TIF) [file pone.0111993.s003.tif]

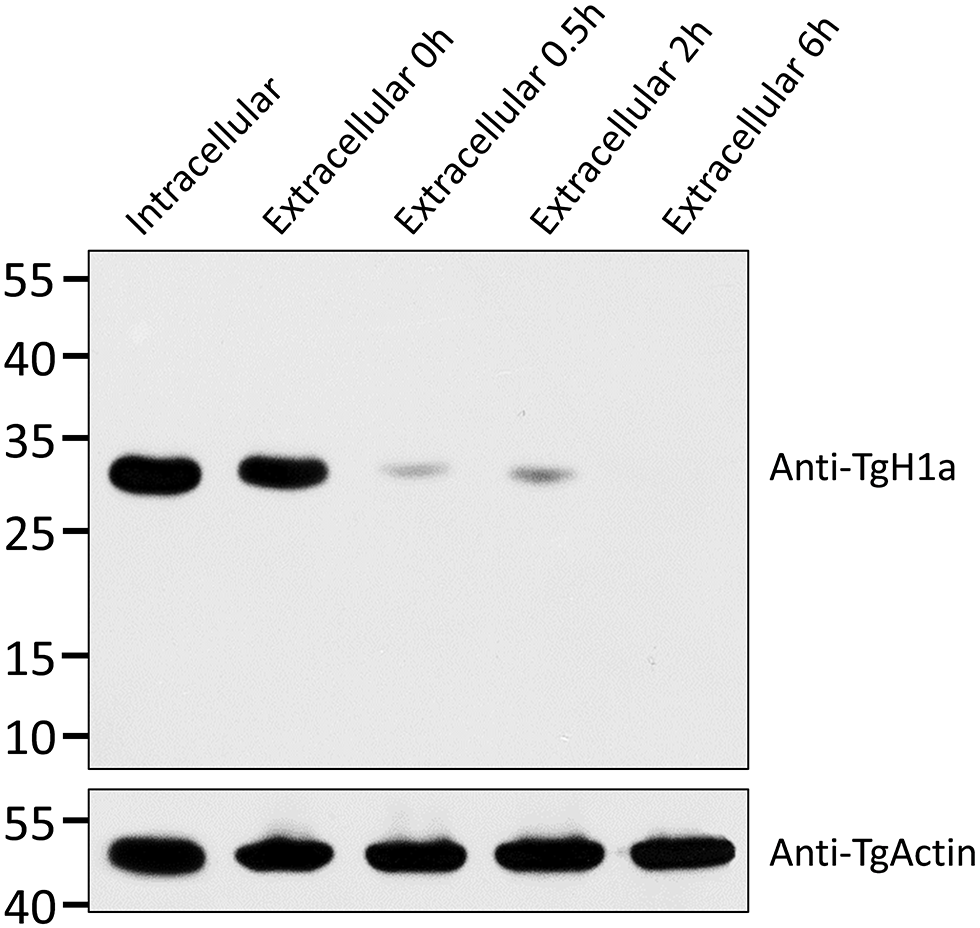

Supplement: Figure S4 — TgHMGB1a constantly express in the intracellular parasites but dramatically decreased in extracellular parasites. The intracellular parasites and freshly released parasites were collected to lysed using RIPA (strong) buffer added cooktail proteinase inhibitor for 30 min on ice, and then analyzed by western blot with anti-TgHMGB1a 4E sera. Meanwhile, freshly released tachyzoites (extracellular 0 h) were incubated additional 0.5, 2 and 6 h in 10% FBS DMEM at 37°C, then centrifuged (800 g, 8 min) and collected the pellets to analyze together with the intracellular parasites. TgActin abs was used to normalize the quantity of per samples. (TIF) [file pone.0111993.s004.tif]

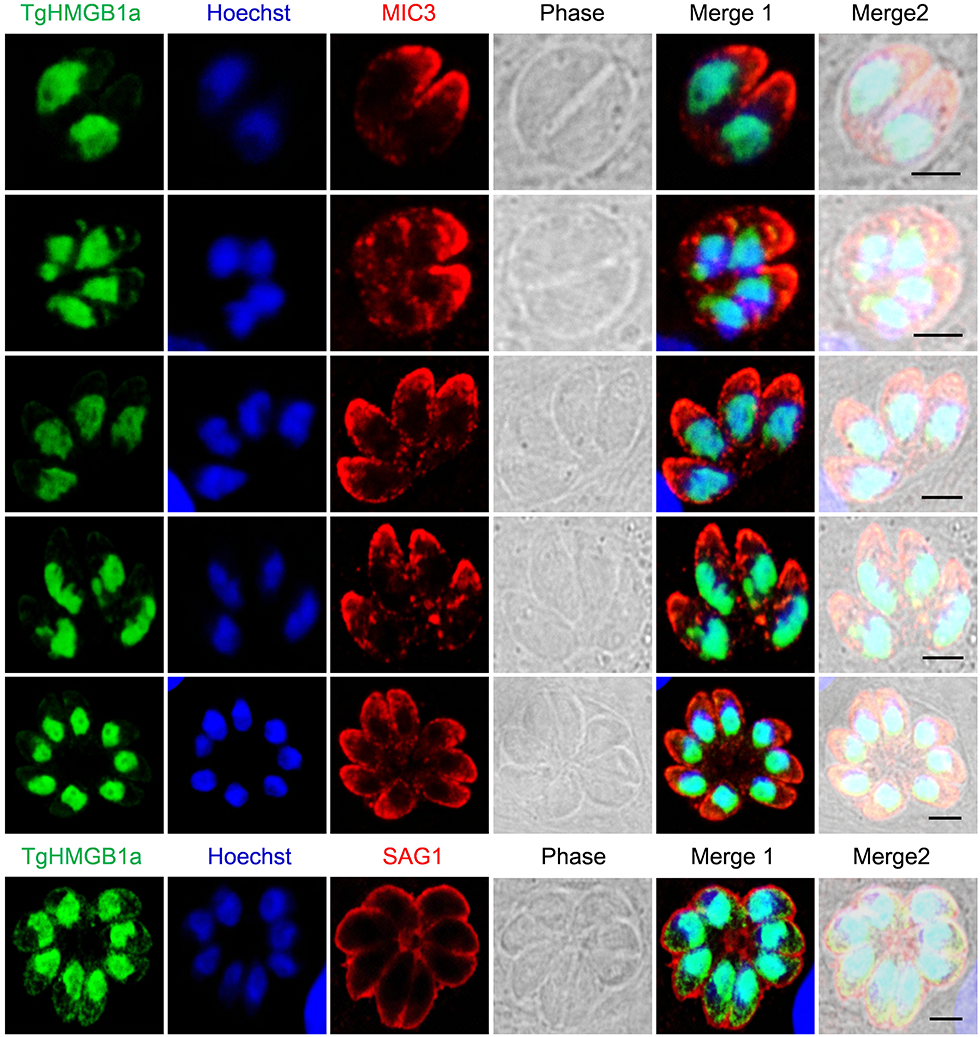

Supplement: Figure S5 — Localization of TgHMGB1a in the intracellular stage. Top panels: TgHMGB1a always concentrated in the parasites nuclear during the course of endodyogeny. Anti-MIC3 antibodies were used as the control (indicated cytoplasm region). Scale bar, 3 µm. Bottom panels: TgHMGB1a is concentrated in the nucleus and less dispersed in the cytoplasm. Anti-SAG1 antibodies were used to label the cytoplasm membrane. Scale bar, 3 µm. (TIF) [file pone.0111993.s005.tif]

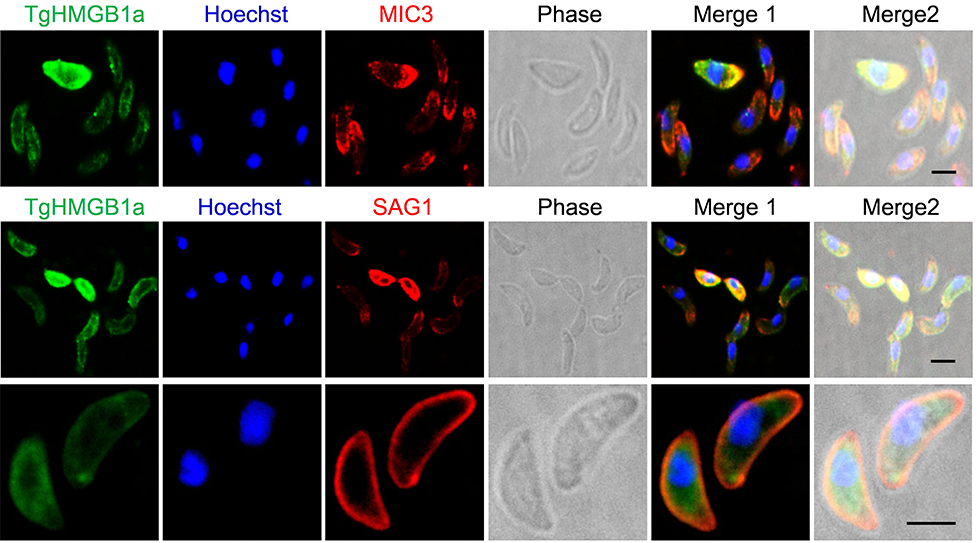

Supplement: Figure S6 — Localization of TgHMGB1a in the extracellular stage. Top panels: TgHMGB1a translocates into cytoplasm when the parasites released to extracellular. Anti-MIC3 antibodies were used as the control. Scale bar, 5 µm. Bottom panels: TgHMGB1a translocates into cytoplasm but not enriched in nucleus when the parasites released to extracellular. Anti-SAG1 antibodies were used to label the cytoplasma membrane. Top panel scale bar is 3 µm and bottom panel scale bar is 3 µm. (TIF) [file pone.0111993.s006.tif]

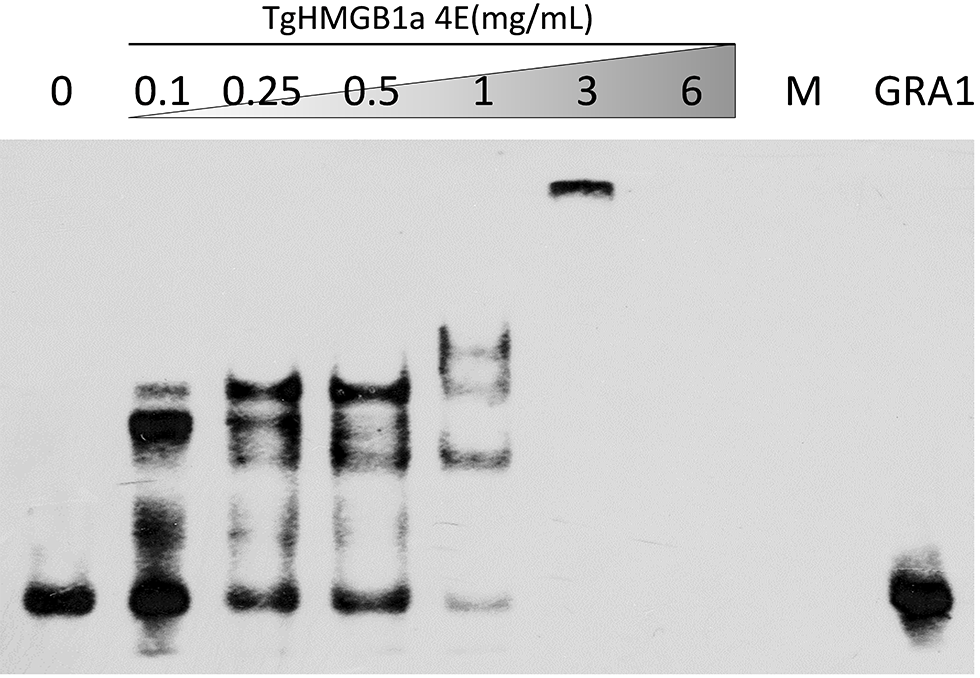

Supplement: Figure S7 — EMSAs with cruciform DNA (4H DNA) and recombinant TgHMGB1a 4E protein or recombinant TgGRA1 protein. Increasing concentrations of TgHMGB1a 4E protein (0.1–6 mg/mL) were incubated with 5 nmol of biotinlabeled 4H DNA for 30 min at 25°C, 2 mg/mL TgGRA1 was used as control. DNA-protein complexes formation were resolved on 6% non-denaturing polyacrylamide gel and transferred to nylon membrane and then detected by Chemiluminescent EMSA Kit. Under identical conditions, only the TgHMGB1a 4E protein can binds cruciform DNA, but not an electrophoretic mobility shift of the 4H probe with TgGRA1 even though in a high concentration. (TIF) [file pone.0111993.s007.tif]

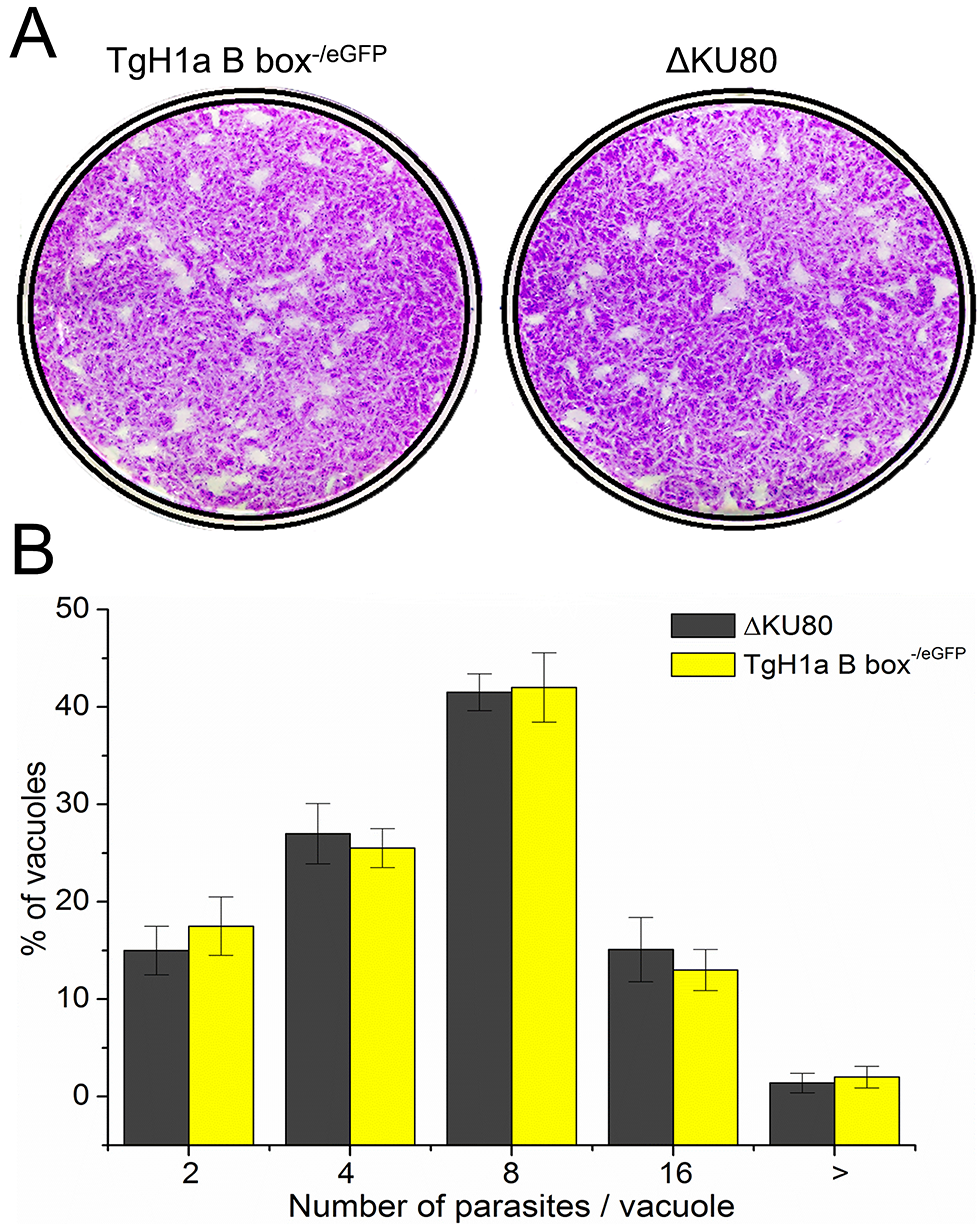

Supplement: Figure S8 — Phenotypic analysis of the TgHMGB1a B box-/eGFP mutant strain. A. Monolayers of HFF were infected with RHΔKU80 or TgHMGB1a B box-/eGFP parasites. Plaques were allowed to form undisturbed and, at 7 days post inoculation, the monolayer was fixed and stained (see Materials and methods). B. An intracellular growth rate assay was carried out after 24 hr of parasite growth. IFA was performed with TgSAG1 abs and the numbers of parasites per vacuole were counted. Each bar indicates the mean ± SD from three independent experiments; n≥150 vacuoles. (TIF) [file pone.0111993.s008.tif]

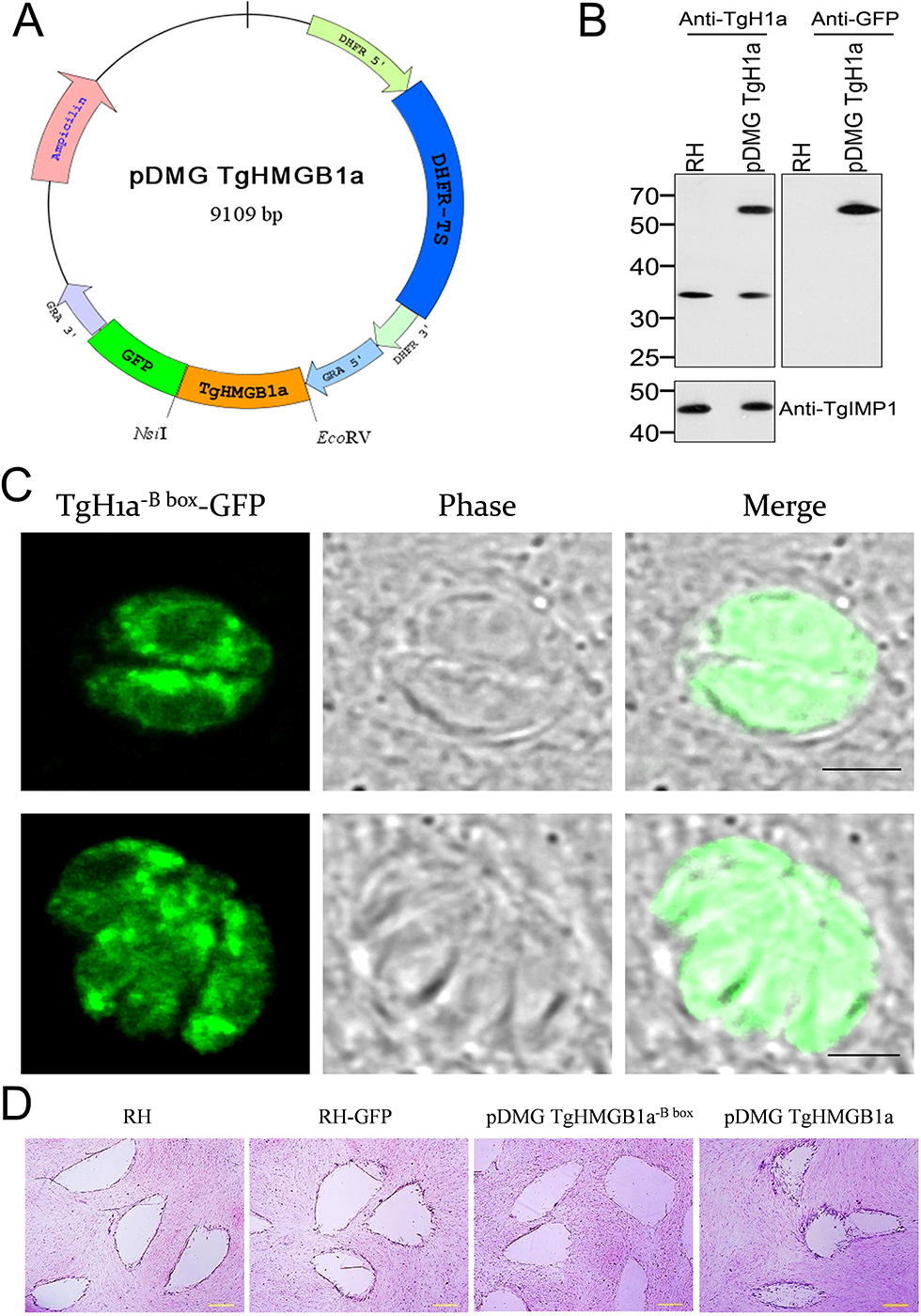

Supplement: Figure S9 — TgHMGB1a overexpression in the RH strain. A. Schematic of the plasmid transfected into the RH strain for overexpression of TgHMGB1a. TgHMGB1a was fused to GFP, which was under the control of the GRA1 promoter. B. TgHMGB1a overexpression was confirmed by western blotting. As expected, transfected clones showed two TgHMGB1a bands (i.e., one is endogenous TgHMGB1a and the other is TgHMGB1a-GFP). When anti-GFP antibodies were used, only the transfected parasite antigens showed specific binding. As an internal control, polyclonal antibodies against T. gondii immune mapped protein 1 (TgIMP1) were used. C. TgHMGB1a-B box-GFP was not concentrated in the nucleus. The plasmid of pDMG TgHMGB1a-B box were transfected into RH strain, and the monoclone were obtained by FCM sorting. B box deficient TgHMGB1a has not localized in nuclear of parasites as showed by the fused GFP, and it likely dispersed in cytoplasm. D. To analyze whether the phenotypes changes were specific to the HMG domain of TgHMGB1a in the overexpress strains, pDMG TgHMGB1a-B box, pDMG TgHMGB1a transfected clones and their parental strains (RH and RH-GFP) were examined by plaque assays. The results showed that only plaques of pDMG TgHMGB1a transfected clones were smaller than their parental strains, demonstrating the HMG domain is involved to the phenotype changes. These pictures were representative of 3 experiments with similar outcomes. Scale bar, 0.2 mm. (TIF) [file pone.0111993.s009.tif]
