# Supplementary material for: A Nuclear Factor of High Mobility Group Box Protein in Toxoplasma gondii
Source: PLoS One. 2014 Nov 4;9(11):e111993. doi: 10.1371/journal.pone.0111993 (PMC4219823; doi:10.1371/journal.pone.0111993)
Supplement: Table S1 — Sequences were used to phylogenetic analysis. (DOCX) [file pone.0111993.s010.docx]

**Table S1.Sequences were used to phylogenetic analysis**

| Name | Database, accession number | Number of HMG boxes |
| --- | --- | --- |
| Homo sapiens HMGB1 | GenBank, NM_002128 | 2 |
| Homo sapiens HMGB2 | GenBank, NP_001124161 | 2 |
| Homo sapiens HMGB3 | GenBank, NM_005342 | 2 |
| Homo sapiens HMGB4 | GenBank, AAH70148 | 2 |
| Mus musculus HMGB1 | GenBank, BC083067 | 2 |
| Mus musculus HMGB2 | GenBank, NM_008252 | 2 |
| Mus musculus HMGB3 | GenBank, NM_008253 | 2 |
| Mus musculus HMGB4 | GenBank, BC061030 | 2 |
| Bos Taurus HMGB1 | GenBank, NM_176612 | 2 |
| Canis lupus familiaris HMGB1 | GenBank, NM_001002937 | 2 |
| Oryctolagus cuniculus HMGB1 | GenBank, DP001022 | 2 |
| Xenopus laevis HMGB1 | GenBank, NM_001087367 | 2 |
| Danio rerio HMGB1 | GenBank, BC045917 | 2 |
| Arabidopsis thaliana HMGB1 | GenBank, AEE78855 | 1 |
| Arabidopsis thaliana HMGB2 | GenBank, NM_101920 | 1 |
| Arabidopsis thaliana HMGB3 | GenBank, NM_001035998 | 1 |
| Arabidopsis thaliana HMGB4 | GenBank, NM_127310 | 1 |
| Saccharomyces cerevisiae NHP6A | GenBank, NM_001184149 | 1 |
| Saccharomyces cerevisiae NHP6B | GenBank, NM_001180058 | 1 |
| Entamoeba histolytica | GenBank, XM_652200 | 1 |
| Plasmodium falciparum HMGB1 | GenBank, XP_001349346 | 1 |
| Plasmodium falciparum HMGB2 | GenBank, XP_001350438 | 1 |
| Leishmania major | EuPathDB, XM_842651 | 1 |
| Schistosoma mansoni | EuPathDB, AY485339 | 2 |
| Trypanosoma brucei brucei | GenBank, XP_843941 | 2 |
| Trypanosoma cruzi | GenBank, XP_810170 | 2 |
| Eimeria necatrix Houghton | ToxoDB, ENH_00063440 | 1 |
| E. tenella strain Houghton | ToxoDB, ETH_00027140 | 1 |
| E. necatrix Houghton | ToxoDB, ENH_00055550 | 1 |
| E. tenella strain Houghton | ToxoDB, ETH_00031100 | 1 |
| E. acervulina Houghton | ToxoDB, EAH_00054690 | 1 |
| Neospora caninum | ToxoDB, NCLIV_024230 | 1 |
| Neospora caninum | ToxoDB, NCLIV_043670 | 1 |
| Neospora caninum | ToxoDB, NCLIV_060790 | 1 |
| Toxoplasma gondii | ToxoDB, TGGT1_210408 | 1 |
| Toxoplasma gondii | ToxoDB, TGGT1_219828 | 1 |
| Toxoplasma gondii | ToxoDB, TGGT1_263720 | 1 |
| Toxoplasma gondii | ToxoDB, TGME49_210408 | 1 |
| Toxoplasma gondii | ToxoDB, TGME49_219828 | 1 |
| Toxoplasma gondii | ToxoDB, TGME49_263720 | 1 |
| Toxoplasma gondii | ToxoDB, TGVEG_210408 | 1 |
| Toxoplasma gondii | ToxoDB, TGVEG_219828 | 1 |
| Toxoplasma gondii | ToxoDB, TGVEG_263720 | 1 |

Data from search in GenBank and EuPathDB and ToxoDB ver.11.
