# Supplementary material for: A Nuclear Factor of High Mobility Group Box Protein in Toxoplasma gondii
Source: PLoS One. 2014 Nov 4;9(11):e111993. doi: 10.1371/journal.pone.0111993 (PMC4219823; doi:10.1371/journal.pone.0111993)
Supplement: Table S2 — Primers used for clone and prokaryotic expression. (DOCX) [file pone.0111993.s011.docx]

**Table S2. Primers used for TgHMGB1a clone and prokaryotic expression**

| Name | 5’-3’ sequence |
| --- | --- |
| TgHMGB1a 4E F | 5' CGGGATCC CACCAAGCTGCAGCCTGCTGCTACC 3' |
| TgHMGB1a 4E R | 5' GCCTCGAG TTATTTGCCACCCTTCTTGTAGGC 3' |
| Tg HMGB1a A F | 5' CGGGATCC ATGTTGTCCATTCTGAAGAATGATC 3' |
| Tg HMGB1a A R | 5' GCAGGCTGCAGCTTGGTGCTTTCCATAGTGTTCCTCAATTGTATTGAC 3' |
| Tg HMGB1a B F | 5’ GTCAATACAATTGAGGAACACTATGGAAAGCACCAAGCTGCAGCCTGC 3’ |
| Tg HMGB1a B R | 5' GCGTCGACTTATTTGCCACCCTTCTTGTAGGC 3' |
| Mice HMGB1 F | 5' CGGGATCCATGGGCAAAGGAGATCCTAAAAAGC 3' |
| Mice HMGB1 R | 5' CCGCTCGAGTTATTCATCATCATCATCTTCTTCTTCATC 3' |
| MH1 A box F | 5' CGGGATCCATGGGCAAAGGAGATCCTAAAAAGC 3' |
| MH1 A box R | 5' CCGCTCGAGTTACTTCTTTTTGGTCTCCCCTTTGG 3' |
| MH1A-TgH1aBAR | 5' GTAGCAGCAGGCTGCAGCTTGGTGCTTCTTTTTGGTCTCCCCTTTGGG 3' |
| MH1A-TgH1aBBF | 5' CCCAAAGGGGAGACCAAAAAGAAGCACCAAGCTGCAGCCTGCTGCTAC 3' |
| MH1 B F | 5' CGC GGATCC TTCAAGGACCCCAATGCACCCAAGAG 3' |
| MH1 B R | 5' CCG CTCGAG TTATTCATCATCATCATCTTCTTCTTCATC 3' |

F, forward; R, reverse
