# Supplementary material for: A Nuclear Factor of High Mobility Group Box Protein in Toxoplasma gondii
Source: PLoS One. 2014 Nov 4;9(11):e111993. doi: 10.1371/journal.pone.0111993 (PMC4219823; doi:10.1371/journal.pone.0111993)
Supplement: Table S3 — Primers used for TgHMGB1a overexpress and B box mutation. (DOCX) [file pone.0111993.s012.docx]

**Table S3. Primers used for TgHMGB1a overexpress and B box mutation**

| Name | 5’-3’ sequence |
| --- | --- |
| pDMG-Tg H1a F | 5' GTCGATATC ATGTTGTCCATTCTGAAGAATGATC 3' |
| pDMG-Tg H1a R | 5' CGCATGCATCCTAGGTTTGCCACCCTTCTTGTAGGCAATC 3' |
| pDMG-Tg H1a^-B^ F | 5' GTCGATATC ATGTTGTCCATTCTGAAGAATGATC 3' |
| pDMG-Tg H1a^-B^ R | 5’ CGCATGCAT CCTAGGGCAGCAGGCTGCAGCTTGGTG 3’ |
| CAT-RFP A F | 5' CCG CTCGAGGTCGACGGTATCGATAAG 3' |
| CAT-RFP A R | 5' CGTCCTCGGTGTTGTCCATGGATCCTCCAGCCCCGCCCTGCCACTCATCG 3' |
| CAT-RFP B F | 5' CGATGAGTGGCAGGGCGGGGCTGGAGGATCCATGGACAACACCGAGGACG 3' |
| CAT-RFP B R | 5' GAGAAGTGAGCACAACGGTGATTAACTACTGGGAGCCGGAGTGGCGGG 3' |
| CAT-RFP C F | 5' CCCGCCACTCCGGCTCCCAGTAGTTAATCACCGTTGTGCTCACTTCTC 3' |
| CAT-RFP C R | 5' CGGGATATCTCTAGTGGATCCCCCTC 3' |
| eGFP KO A F | 5' GAGCCTAGGGTTCGATCCATTACGCGGGAAACAAC 3' |
| eGFP KO A R | 5' CCTTGCTCACCATGGATCCTCCCGCGTTGGGGTCCTTCTTCGCCTTGGTCTTTTTG 3' |
| eGFP KO B F | 5' CAAAAAGACCAAGGCGAAGAAGGACCCCAACGCGGGAGGATCCATGGTGAGCAAGG 3' |
| eGFP KO B R | 5' CCTTAATTAATCACTTGTACAGCTCGTCCATGCCG 3' |

F, forward; R, reverse
