# Supplementary material for: A Nuclear Factor of High Mobility Group Box Protein in Toxoplasma gondii
Source: PLoS One. 2014 Nov 4;9(11):e111993. doi: 10.1371/journal.pone.0111993 (PMC4219823; doi:10.1371/journal.pone.0111993)
Supplement: Table S4 — Primers used for qRT-PCR. (DOCX) [file pone.0111993.s013.docx]

**Table S4. Primers used for qRT-PCR**

| Name | 5’-3’ sequence | Products (bp) |
| --- | --- | --- |
| TgActin F | 5' GACCTTACCGAGTACATGATGAAG 3' | 195 |
| TgActin R | 5' CCATCGGGCAATTCATAGGAC 3' |  |
| TgH1b F | 5' GGTCTGTTGACGGTGGATTTG 3' | 183 |
| TgH1b R | 5' CGCCGACGGCTCATGCGCTCTC 3' |  |
| TgH1c F | 5' CTGAAACACATTTGTCTCTTCAACTCG 3' | 181 |
| TgH1c R | 5' TCCTTCTTCTTTCTGCCGTTTCGCCTC 3' |  |
| TgROP18 F | 5' ACCGATGAGACTATGCTTGACTTGC 3' | 160 |
| TgROP18 R | 5' CAATCACGACCTCTGTGGATGCTG 3' |  |
| TgROP16 F | 5' CTGAACAAGCGGCGGCTGAAGTAG 3' | 207 |
| TgROP16 R | 5' TGCTGCGTGGTGGAAGATGCGAGGGTG 3' |  |
| TgToxofilin F | 5' TTTTCTGCTGCTCATTACCGTC 3' | 131 |
| TgToxofilin R | 5' CTCTCAAATCTTCCCCTTTTCG 3' |  |
| TgMIC3 F | 5' CAGGAGACGCAACTCTGTGCTATC 3' | 201 |
| TgMIC3 R | 5' TGAGCGAACTGCTGCAAAAAATCC 3' |  |
| TgPLP F | 5’ GATAAACAGTATCCTGGACTTCGAG 3’ | 148 |
| TgPLP R | 5’ CCATAACATAACCAAGGACAATGGA 3’ |  |
| TgProfilin F | 5' GTACAACGACTGCACCTTCGACATCAC 3' | 138 |
| TgProfilin R | 5' GTCCTGCTGTTTCCCTTGTCCTGTTCC 3' |  |
| TgGRA7 R | 5' TCCGACGCTGAAGTGACTGACGACAAC 3' | 150 |
| TgGRA7 R | 5' GGCAAAATACGATGCACCCATACCAAC 3' |  |

F, forward; R, reverse
