# Supplementary material for: A Nuclear Factor of High Mobility Group Box Protein in Toxoplasma gondii
Source: PLoS One. 2014 Nov 4;9(11):e111993. doi: 10.1371/journal.pone.0111993 (PMC4219823; doi:10.1371/journal.pone.0111993)
Supplement: Table S5 — Primers of target gene promoters used for ChIP-qPCR. (DOCX) [file pone.0111993.s014.docx]

**Table S5. Primers of target gene promoters used to ChIP-qPCR analysis**

| Name | 5’-3’ sequence | Products (bp) |
| --- | --- | --- |
| ROP18-QC 5’ | 5’ GCACACCACCATGCTGTCTA 3’ | 118 |
| ROP18-QC 3’ | 5’ GTTCCTGGCACCCTTTTGTA 3’ |  |
| ROP16-QC 5’ | 5' ACGTCTGACCACGCATATTAGG 3' | 182 |
| ROP16-QC 3’ | 5' AAACAAGATCACAGGCAGTTTC 3' |  |
| Toxofilin-QC 5’ | 5' AGCATACCAGACCCGAAGGCAG 3' | 182 |
| Toxofilin-QC 5’ | 5' CGCAATAACTAGCTGGCAGAAG 3' |  |
| MIC3-QC 5’ | 5' CCTTCGTTCACCGGTACACCTC 3' | 179 |
| MIC3-QC 3’ | 5' GCACTCTCGACGAGCTGCGACC 3' |  |
| PLP1-QC 5’ | 5' CTCCCGTCCATTTATTTACCGT 3' | 242 |
| PLP1-QC 3’ | 5' CGATTGTGAAAGGAGGCCAAAC 3' |  |
| GRA7-QC 5’ | 5' CCTGATTTCGTTTACCATTGAC 3' | 263 |
| GRA7-QC 3’ | 5' CCGCAGTAATCCCTTTTAATAC 3' |  |
| Profilin-QC 5’ | 5' GTACGCGTGACGGTTTTAAGGC 3' | 239 |
| Profilin-QC 3’ | 5' GAATGGAAACAAGCCGCGAAAG 3' |  |
