# Supplementary material for: A Nuclear Factor of High Mobility Group Box Protein in Toxoplasma gondii
Source: PLoS One. 2014 Nov 4;9(11):e111993. doi: 10.1371/journal.pone.0111993 (PMC4219823; doi:10.1371/journal.pone.0111993)
Supplement: Table S6 — Repertoire of high mobility group box proteins in three genotypes T.gondii . (DOCX) [file pone.0111993.s015.docx]

**Table S5.Repertoire of high mobility group box proteins in three genotypes *T.gondii***

| ToxoDB  accession number | Chromosome | Protein coding length | Number of HMG boxes |
| --- | --- | --- | --- |
| TGGT1_210408 | TGGT1_chr IX | 94 | 1 |
| TGGT1_219828 | TGGT1_chr XII | 98 | 1 |
| TGGT1_263720 | TGGT1_chr VIIb | 145 | 1 |
| TGME49_210408 | TGME49_chr IX | 94 | 1 |
| TGME49_219828 | TGME49_chr XII | 98 | 1 |
| TGME49_263720 | TGME49_chr VIIb | 145 | 1 |
| TGVEG_210408 | TGVEG_chr IX | 94 | 1 |
| TGVEG_219828 | TGVEG_chr XII | 98 | 1 |
| TGVEG_263720 | TGVEG_chr VIIb | 145 | 1 |

Data from blast analysis using mice Mus musculus high mobility group box 1 (GenBank: BC083067.1) in ToxoDB version 11.0.
