# Supplementary material for: A Nuclear Factor of High Mobility Group Box Protein in Toxoplasma gondii
Source: PLoS One. 2014 Nov 4;9(11):e111993. doi: 10.1371/journal.pone.0111993 (PMC4219823; doi:10.1371/journal.pone.0111993)
Supplement: Data S1 — Completed ARRIVE checklist for animal infection experiments. (DOCX) [file pone.0111993.s016.docx]

**Title: Virulence study of two transgenic parasites (TgHMGB1a overexpress and B box deficient strains)**

**Abstract** TgHMGB1a is a nuclear factor, which involved to gene transcription in *T.gondii* development. We constructed two transgenic parasites, one is the TgHMGB1a overexpress, and another is deficient of functional domain (B box). To determine whether TgHMGB1a mutant influence the parasitic virulence to mice, female Balb/c mice were infected intraperitoneally (i.p), and monitored survivals. We found the survival of Balb/c mice infected with TgHMGB1a B box^-/eGFP^ or RHΔKU80 strains were not significantly different (**Figure 7D**). However, mice infected with the TgHMGB1a overexpression showed a significantly delayed time to death (3 to 5 days) compared to its parental RH or RH-GFP strains, while the RH and RH-GFP strains showed almost equivalent virulence (**Figure 7C**).

**Introduction**

Toxoplasma is an obligate intracellular protozoan, and can cause opportunistic disease in various animals and humans [1]. There are three *T. gondii* genotypes, types I, II, and III, which have different growth characteristics [2] and cause variable levels of virulence in mice [3,4]. Virulence difference in the three types is a result of many virulence effector proteins (including ROP18, ROP5 and ROP17 and so on). Parasites developmental transitions are accompanied by major changes in gene expression [5], and the control mechanisms for parasite proliferation (replication and differentiation) may be regulated by the cell cycle [6,7] and the micro-environments around the parasites [8], which might also affect virulence. TgHMGB1a is nuclear factor which involved to transcription, but the affection to parasitic virulence in mice is poorly understood. To reveal whether TgHMGB1a mutant influence the parasitic virulence in mice, we examined the mice survival after infected with TgHMGB1a overexpress and TgHMGB1a B box deficient parasites.

**Methods**

***Ethical statement***

All experiments with animals in this study were performed in strict accordance with the recommendations in the Guide for the Care and Use of Laboratory Animals of the Ministry of Science and Technology of China. Formal animal ethics of all experimental procedures were approved by the Institutional Animal Care and Use Committee of China Agricultural University (The certificate of Beijing Laboratory Animal employee, Approval No: 18049). All efforts were made to minimize animal suffering.

***Study design***

Part I (Figure 6 C) virulence study of TgHMGB1a overexpress strain

| Parasites strain | Infection dose | Mice per dose | Monitoring item |
| --- | --- | --- | --- |
| RH-TgHMGB1a overexpress | 10^2^ | 5 | Survival time (St) |
|  | 10^3^ | 5 | St |
|  | 10^4^ | 5 | St |
| RH (parental strain) | 10^2^ | 5 | St |
|  | 10^3^ | 5 | St |
|  | 10^4^ | 5 | St |
| RH-GFP (GFP control) | 10^2^ | 5 | St |
|  | 10^3^ | 5 | St |
|  | 10^4^ | 5 | St |

Part II (Figure 6 D) virulence study of TgHMGB1a B box^-/eGFP^ strain

| Parasites strain | Infection dose | Mice per dose | Monitoring item |
| --- | --- | --- | --- |
| TgHMGB1a B box^-/eGFP^ | 10^2^ | 5 | Survival time (St) |
|  | 10^3^ | 5 | St |
|  | 10^4^ | 5 | St |
| RHΔKU80 (parental strain) | 10^2^ | 5 | St |
|  | 10^3^ | 5 | St |
|  | 10^4^ | 5 | St |

This experiment design was carried out three times independently.

***Experimental animals***

The mice were all 6- to 8-week-old female Balb/c mice (The Center for Experimental Animals, Beijing) and randomly divided into five mice per group (a cage). All the animals were housed under specific pathogen-free conditions for 7 days before manipulation. Rodent laboratory chow and tap water were provided ad libitum and maintained under controlled conditions with a temperature of 24±1 °C, humidity of 50±10%, and a 12:12 h light/dark cycle. All procedures were in strict accordance with the PR China legislation on use. All efforts were made to minimize animal suffering.

**Experimental procedures**

***Cell culture and parasites maintain***

HFF (human foreskin fibroblasts) were cultured in complete DMEM, as described previously [9]. RH-TgHMGB1a overexpress, RH and RH-GFP tachyzoites, and TgHMGB1a B box^-/eGFP^ and RHΔKU80 strains were maintained in vitro by serial passage on confluent HFF monolayers in DMEM containing 25 mM glucose and 4 mM glutamine supplemented with 10% fetal bovine serum (FBS, Gibco, USA), and incubated at 37 ºC with 5% CO_2_ in a humidified incubator.

***Mice inoculation***

Freshly egressed parasites were collected, purified (using 5 μm filter), washed twice in PBS and counted using a hemocytometer. Ultimately, parasites were diluted in pre-warmed PBS and inoculated into mice by intraperitoneal (i.p.) injection at doses indicated in **study design** (both of the Part I and Part II). And, PBS was injected as control (5 mice). Five mice were housed in a cage (per dose) under specific pathogen-free conditions. Food and water were freely available throughout the experiments.

***Animal monitoring***

All the infected mice were monitored three times a day (every 8 hours) for clinical signs and mortality until there were no remaining survivors.

***Humane Endpoints***

The following are general humane endpoints for rodents that require euthanasia in this study.

1. The inability to reach food or water for more than 24 hours.

2. 20% decrease in normal body weight.

***Euthanasia for mice who reached to the humane endpoints***

If the mice reached to the humane endpoints described above, mice will be anesthetized by subcutaneous injection of Atropine (0.02 mg/kg), and then were humanely euthanized by cervical dislocation. The humane sacrificed time were recorded as death time of mice.

**Results**

The humane euthanasia time were recorded as time of death of the experimental mice.

Table 1 Death time of mice in the first infection experiment (26 October 2013)

| Group | | Date of death (six mice per dose) | | | | |
| --- | --- | --- | --- | --- | --- | --- |
| RH | 10^2^ | 6 Nov. | 6 Nov. | 7 Nov. | 7 Nov. | 8 Nov. |
|  | 10^3^ | 4 Nov. | 5 Nov. | 5 Nov. | 6 Nov. | 6 Nov. |
|  | 10^4^ | 2 Nov. | 3 Nov. | 3 Nov. | 4 Nov. | 4 Nov. |
| RH-GFP | 10^2^ | 6 Nov. | 7 Nov. | 7 Nov. | 7 Nov. | 8 Nov. |
|  | 10^3^ | 3 Nov. | 4 Nov. | 4 Nov. | 4 Nov. | 6 Nov. |
|  | 10^4^ | 3 Nov. | 3 Nov. | 4 Nov. | 4 Nov. | 5 Nov. |
| pDMG TgHMGB1a | 10^2^ | 8 Nov. | 9 Nov. | 10 Nov. | 12 Nov. | 12 Nov. |
|  | 10^3^ | 8 Nov. | 8 Nov. | 9 Nov. | 9 Nov. | 10 Nov. |
|  | 10^4^ | 6 Nov. | 6 Nov. | 7 Nov. | 7 Nov. | 9 Nov. |
| PBS | 100μL | —— | —— | —— | —— | —— |
| ΔKU80 | 10^2^ | 6 Nov. | 7 Nov. | 9 Nov. | 9 Nov. | 10 Nov. |
|  | 10^3^ | 5 Nov. | 5 Nov. | 6 Nov. | 6 Nov. | 7 Nov. |
|  | 10^4^ | 4 Nov. | 4 Nov. | 5 Nov. | 5 Nov. | 7 Nov. |
| TgHMGB1a B box^-/eGFP^ | 10^2^ | 7 Nov. | 7 Nov. | 7 Nov. | 10 Nov. | 11 Nov. |
|  | 10^3^ | 5 Nov. | 5 Nov. | 6 Nov. | 7 Nov. | 7 Nov. |
|  | 10^4^ | 4 Nov. | 5 Nov. | 6 Nov. | 6 Nov. | 6 Nov. |

Table 2 Death time of mice in the secondary infection experiment (26 November 2013)

| Group | | Date of death (six mice per dose) | | | | |
| --- | --- | --- | --- | --- | --- | --- |
| RH | 10^2^ | 5 Dec. | 5 Dec. | 5 Dec. | 7 Dec. | 7 Dec. |
|  | 10^3^ | 4 Dec. | 4 Dec. | 5 Dec. | 6 Dec. | 7 Dec. |
|  | 10^4^ | 3 Dec. | 3 Dec. | 4 Dec. | 4 Dec. | 6 Dec. |
| RH-GFP | 10^2^ | 5 Dec. | 7 Dec. | 7 Dec. | 8 Dec. | 8 Dec. |
|  | 10^3^ | 3 Dec. | 4 Dec. | 5 Dec. | 6 Dec. | 6 Dec. |
|  | 10^4^ | 2 Dec. | 3 Dec. | 4 Dec. | 5 Dec. | 5 Dec. |
| pDMG TgHMGB1a | 10^2^ | 9 Dec. | 9 Dec. | 12 Dec. | 12 Dec. | 13 Dec. |
|  | 10^3^ | 8 Dec. | 9 Dec. | 10 Dec. | 10 Dec. | 11 Dec. |
|  | 10^4^ | 6 Dec. | 6 Dec. | 7 Dec. | 8 Dec. | 9 Dec. |
| PBS | 100μL | —— | —— | —— | —— | —— |
| ΔKU80 | 10^2^ | 5 Dec. | 7 Dec. | 7 Dec. | 9 Dec. | 10 Dec. |
|  | 10^3^ | 5 Dec. | 5 Dec. | 6 Dec. | 6 Dec. | 8 Dec. |
|  | 10^4^ | 3 Dec. | 4 Dec. | 5 Dec. | 6 Dec. | 6 Dec. |
| TgHMGB1a B box^-/eGFP^ | 10^2^ | 7 Dec. | 7 Dec. | 8 Dec. | 10 Dec. | 10 Dec. |
|  | 10^3^ | 5 Dec. | 5 Dec. | 6 Dec. | 7 Dec. | 7 Dec. |
|  | 10^4^ | 4 Dec. | 5 Dec. | 5 Dec. | 6 Dec. | 7 Dec. |

Table 3 Death time of mice in the third infection experiment (22 December 2013)

| Group | | Date of death (six mice per dose) | | | | |
| --- | --- | --- | --- | --- | --- | --- |
| RH | 10^2^ | 3 Jan.2014 | 4 Jan. | 5 Jan. | 6 Jan. | 6 Jan. |
|  | 10^3^ | 3 Jan. | 3 Jan. | 3 Jan. | 4 Jan. | 4 Jan. |
|  | 10^4^ | 1 Jan. | 2 Jan. | 2 Jan. | 3 Jan. | 4 Jan. |
| RH-GFP | 10^2^ | 4 Jan. | 4 Jan. | 4 Jan. | 5 Jan. | 6 Jan. |
|  | 10^3^ | 3 Jan. | 4 Jan. | 4 Jan. | 4 Jan. | 6 Jan. |
|  | 10^4^ | 2 Jan. | 2 Jan. | 3 Jan. | 3 Jan. | 3 Jan. |
| pDMG TgHMGB1a | 10^2^ | 6 Jan. | 9 Jan. | 9 Jan. | 10 Jan. | 10 Jan. |
|  | 10^3^ | 6 Jan. | 6 Jan. | 7 Jan. | 7 Jan. | 9 Jan. |
|  | 10^4^ | 5 Jan. | 6 Jan. | 6 Jan. | 6 Jan. | 7 Jan. |
| PBS | 100μL | —— | —— | —— | —— | —— |
| ΔKU80 | 10^2^ | 4 Jan. | 4 Jan. | 4 Jan. | 5 Jan. | 7 Jan. |
|  | 10^3^ | 2 Jan. | 3 Jan. | 5 Jan. | 5 Jan. | 5 Jan. |
|  | 10^4^ | 1 Jan. | 2 Jan. | 2 Jan. | 4 Jan. | 4 Jan. |
| TgHMGB1a B box^-/eGFP^ | 10^2^ | 5 Jan. | 5 Jan. | 5 Jan. | 7 Jan. | 7 Jan. |
|  | 10^3^ | 4 Jan. | 4 Jan. | 5 Jan. | 6 Jan. | 7 Jan. |
|  | 10^4^ | 2 Jan. | 3 Jan. | 3 Jan. | 4 Jan. | 4 Jan. |

Three independently experiments indicated similar results, so, **Figure 6 C and D** representative the 3 experiments with similar outcomes. TgHMGB1a B box^-/eGFP^ or RHΔKU80 strains were not significantly different (**Figure 6D**) in the virulence to mice. However, mice infected with the TgHMGB1a overexpression showed a significantly delayed time to death (3 to 5 days) compared to its parental RH and RH-GFP strains, while the RH and RH-GFP strains showed almost equivalent virulence (**Figure 6C**).

**Discussion**

Acute virulence to mice was the most difference of the three genotypes *Toxoplasma gondii*. These results indicated that TgHMGB1a may be involved to the virulence which might related to the transcription regulation. And according to the outcomes, TgHMGB1a should be a negative effect on the virulence, but HMGB1 commonly plays an active role in promotes transcription. Whether are they contradictory each other? How to interpret it? And see more discussions in the text. More and further investigations are needed to reveal and confirm the functions of TgHMGB1 proteins, and verify the relation between the transcription regulation by TgHMGB1 and parasitic virulence.

**References:**

1. Morisaki JH, Heuser JE, Sibley LD (1995) Invasion of Toxoplasma gondii occurs by active penetration of the host cell. J Cell Sci 108 ( Pt 6): 2457-2464.

2. Appleford PJ, Smith JE (1997) Toxoplasma gondii: the growth characteristics of three virulent strains. Acta Trop 65: 97-104.

3. Sibley LD, Ajioka JW (2008) Population structure of Toxoplasma gondii: clonal expansion driven by infrequent recombination and selective sweeps. Annu Rev Microbiol 62: 329-351.

4. Howe DK, Sibley LD (1995) Toxoplasma gondii comprises three clonal lineages: correlation of parasite genotype with human disease. J Infect Dis 172: 1561-1566.

5. Gaji RY, Behnke MS, Lehmann MM, White MW, Carruthers VB (2011) Cell cycle-dependent, intercellular transmission of Toxoplasma gondii is accompanied by marked changes in parasite gene expression. Mol Microbiol 79: 192-204.

6. Dubey JP (1998) Advances in the life cycle of Toxoplasma gondii. Int J Parasitol 28: 1019-1024.

7. Dzierszinski F, Nishi M, Ouko L, Roos DS (2004) Dynamics of Toxoplasma gondii differentiation. Eukaryot Cell 3: 992-1003.

8. Singh U, Brewer JL, Boothroyd JC (2002) Genetic analysis of tachyzoite to bradyzoite differentiation mutants in Toxoplasma gondii reveals a hierarchy of gene induction. Mol Microbiol 44: 721-733.

9. Gaskell EA, Smith JE, Pinney JW, Westhead DR, McConkey GA (2009) A unique dual activity amino acid hydroxylase in Toxoplasma gondii. PLoS One 4: e4801.

Result of statistical analyze the survival between the RH and pDMG TgHMGB1a transfected strain

**Group statistics (**Independent samples T test, SPSS 18.0**)**

|  | Groups | N | Mean | Std. Deviation | Std. Error Mean |
| --- | --- | --- | --- | --- | --- |
| Dose:10^2^ | RH | 15 | 12.0667 | 1.43759 | .37118 |
|  | pDMG TgHMGB1a | 15 | 14.9333 | 1.79151 | .46257 |
| Dose:10^3^ | RH | 15 | 11.0000 | 1.25357 | .32367 |
|  | pDMG TgHMGB1a | 15 | 13.8000 | 1.01419 | .26186 |
| Dose:10^4^ | RH | 15 | 7.9333 | 1.03280 | .26667 |
|  | pDMG TgHMGB1a | 15 | 9.4667 | 1.72654 | .44579 |

**Independent Samples Test**

|  | | Levene’s Test for Equality of Variances | | t test for equality of means | | | | | | |
| --- | --- | --- | --- | --- | --- | --- | --- | --- | --- | --- |
|  |  | F | Sig. | t | df | Sig.  (2-Tailed) | Mean Difference | Std. Error Difference | 95 % Confidence Interval of the Difference | |
|  |  |  |  |  |  |  |  |  | lower | upper |
| Dose 10^2^ | Equal Variance assumed | 1.670 | .207 | -4.834 | 28 | 4.3722364718423755 E-5 | -2.86667 | .59308 | -4.08154 | -1.65179 |
|  | Equal Variance not assumed |  |  | -4.834 | 26.745 | 4.873388507575175 E-5 | -2.86667 | .59308 | -4.08411 | -1.64922 |
| Dose 10^3^ | Equal Variance assumed | .374 | .546 | -6.725 | 28 | 2.6610208744865226 E-7 | -2.80000 | .41633 | -3.65282 | -1.94718 |
|  | Equal Variance not assumed |  |  | -6.725 | 26.830 | 3.3117435904937557 E-7 | -2.80000 | .41633 | -3.65450 | -1.94550 |
| Dose 10^4^ | Equal Variance assumed | 5.514 | .026 | -2.952 | 28 | 0.006327047374981957 | -1.53333 | .51946 | -2.59740 | -.46926 |
|  | Equal Variance not assumed |  |  | -2.952 | 22.882 | 0.0071791319825432725 | -1.53333 | .51946 | -2.60823 | -.45844 |

P<0.001 be consider as significant difference.
